# Improved interval methods for solving circle packing problems in the unit square

**DOI:** 10.1007/s10898-021-01086-z

**Published:** 2021-09-29

**Authors:** Mihály Csaba Markót

**Affiliations:** Faculty of Mathematics, Wolfgang Pauli Institute, University of Vienna, Oskar-Morgenstern-Platz 1, 1090 Vienna, Austria

**Keywords:** Interval arithmetic, Global optimization, Branch and bound, Circle packing, Optimality proof, 52C15, 52C26, 65G30, 65K05, 90C30

## Abstract

In this work computer-assisted optimality proofs are given for the problems of finding the densest packings of 31, 32, and 33 non-overlapping equal circles in a square. In a study of 2005, a fully interval arithmetic based global optimization method was introduced for the problem class, solving the cases 28, 29, 30. Until now, these were the largest problem instances solved on a computer. Using the techniques of that paper, the estimated solution time for the next three cases would have been 3–6 CPU months. In the present paper this former method is improved in both its local and global search phases. We discuss a new interval-based polygon representation of the core local method for eliminating suboptimal regions, which has a simpler implementation, easier proof of correctness, and faster behaviour than the former one. Furthermore, a modified strategy is presented for the global phase of the search, including improved symmetry filtering and tile pattern matching. With the new method the cases $n=31,32,33$ have been solved in 26, 61, and 13 CPU hours, giving high precision enclosures for all global optimizers and the optimum value. After eliminating the hardware and compiler improvements since the former study, the new proof technique became roughly about 40–100 times faster than the previous one. In addition, the new implementation is suitable for solving the next few circle packing instances with similar computational effort.

## Introduction

In this paper we are dealing with optimal (densest) packings of equal circles in a unit square. During the last decades this problem class attracted the attention of many mathematicians and computer scientists. Although the problem has a very simple mathematical formulation, in many cases it is very challenging to find and prove the optimality of a packing configuration. Actually for $n\ge 28$ a whole ‘cookbook’ of various mathematical and numerical techniques is required to tackle the problems.

The paper is organized as follows. In Sect. 2 we review some possible problem models and the history of solving instances of the problem class. In Sects. 3 and 4 we briefly introduce the basics of interval arithmetic calculations and the interval branch–and–bound framework used in this study. In Sect. 5 we discuss the key local elimination procedure that uses a new, mathematically rigorous computer representation of convex polygons. In Sect. 6 we introduce techniques to speed up the global search phase of the optimality proofs. In Sect. 7 we detail the solution process for the problem instances $n=31,32,33$. In Sect. 8 we summarize the main achievements of the paper.

## Problem statement and history

The informal description of the considered *circle packing* problem is the following: *place a given number n of equal circles without overlapping into a unit square, maximizing the diameter of the circles*. This problem is known (see, e.g. [1]) to be equivalent to the following *point packing* problem: *place a given number n of points into the unit square, maximizing the minimal distance between the pairs of points*. That is, there is a bijective mapping (based on simple geometric transformations) between the set of optimal solutions of the problems of packing *n* circles and *n* points. Therefore we consider the simpler point packing problem:

1 $$\begin{aligned}&\mathrm{maximize} \min _{1 \le i < j \le n} \sqrt{{(x_{i}-x_{j})}^2 + {(y_{i}-y_{j})}^2} ,\nonumber \\&\quad \mathrm{s.t.\quad }0 \le x_i, y_i \le 1 , \qquad i = 1,2, \dots ,n, \end{aligned}$$

where the unit square is $[0,1]^2$, and the *i*th point is located at $(x_i,y_i)$. The integer $n\ge 2$ is a parameter of the problem class, thus, one can refer to a particular point packing problem instance by specifying *n*.

Since the square root function is strictly monotone, in practice we solve the problem of maximizing

2 $$\begin{aligned}&f_n: {\mathbb {R}}^{2n} \rightarrow \mathbb {R},\quad f_n(x,y) = \min _{1 \le i < j \le n} {(x_{i}-x_{j})}^2 + {(y_{i}-y_{j})}^2,\nonumber \\&\mathrm{s.t.\quad }0 \le x_i, y_i \le 1 , \qquad i = 1,2, \dots ,n, \end{aligned}$$

saving the evaluation of the square root. In the sequel we will use the shorthand notation $s_{ij} = {(x_{i}-x_{j})}^2 + {(y_{i}-y_{j})}^2$ for the *squared* distance between the *i*th and *j*th point.

Up to now, only the optimal packings of $2,\dots ,9, 14, 16, 25$, and 36 circles have been proved in a theoretical way. On the other hand, computer-assisted optimality proofs exist for $n\le 20$ [2–4], for $21 \le n \le 27$ [5], and for $28 \le n \le 30$ [6]. The first two of these computer approaches use floating point arithmetic and bound rounding errors only during the geometric steps of the algorithms. The third approach by M.C. Markót and T. Csendes, in contrast, presents a fully interval arithmetic based procedure, providing interval enclosures of both the possible optimizers and the optimum values with high accuracy. The required CPU time for solving the cases $n=28,29,30$ was about 53, 50, and 21 hours, resp., on an at that time decent PC desktop architecture. The number of so-called tile combinations to be checked during the global search (a good indicator of the complexity of the optimality proof) is $$\left( {\begin{array}{c}42\\ 28\end{array}}\right) $$, $$\left( {\begin{array}{c}42\\ 29\end{array}}\right) $$, and $$\left( {\begin{array}{c}42\\ 30\end{array}}\right) $$, resp., for these instances. The next three instances $n=31,32,33$ instead require the processing of $$\left( {\begin{array}{c}48\\ 31\end{array}}\right) $$, $$\left( {\begin{array}{c}48\\ 32\end{array}}\right) $$, and $$\left( {\begin{array}{c}48\\ 33\end{array}}\right) $$ combinations. Thus, the case $n=31$ (resp., 32, 33) requires about 100 times more processing effort than the case $n=28$ (resp., 29, 30); see Sect. 6 for a detailed calculation. That is, with the method of [6], the estimated solution time would be 3–6 CPU *months* for $n=31,32,33$. The goal of the present paper is to improve the method of [6], and solve the cases $n=31, 32, 33$ again with reasonable computational effort.

## Interval analysis

As mentioned above, the proof method uses interval computations to produce reliable numerical solutions with mathematical correctness. Below we give only a very brief survey on the basic interval definitions and properties; for more details we refer to, e.g., [7–9].

The set of compact *interval*s is denoted by $\mathbb {I}$, where ${\varvec{a}}=[{\mathrm{inf}}{({\varvec{a}})},{\mathrm{sup}}{({\varvec{a}})}]= \{a \in \mathbb {R}\ |\ {\mathrm{inf}}{({\varvec{a}})} \le a \le {\mathrm{sup}}{({\varvec{a}})} \}$ for all ${\varvec{a}}\in \mathbb {I}$. Here ${\mathrm{inf}}{({\varvec{a}})},{\mathrm{sup}}{({\varvec{a}})}\in \mathbb {R}$ denote the *lower bound* (infimum) and the *upper bound* (supremum) of ${\varvec{a}}$, respectively. If ${\mathrm{inf}}{({\varvec{a}})}={\mathrm{sup}}{({\varvec{a}})}$, we call ${\varvec{a}}$ a *point interval*. The *width* of an interval is defined by $w({\varvec{a}}) := {\mathrm{sup}}{({\varvec{a}})}-{\mathrm{inf}}{({\varvec{a}})}$. For a given set of real numbers $D \subseteq \mathbb {R}$, ${\mathbb {I}}(D)$ denotes the set of all intervals in *D*.

The real *arithmetic operations* can be extended for intervals by applying the general set theoretic definition ${\varvec{a}}\circ {\varvec{b}}:= \{ a \circ b \ |\ a \in {\varvec{a}},\ b\in {\varvec{b}}\}$.

Let $f : D \subseteq \mathbb {R}\rightarrow \mathbb {R}$ be a real *elementary function* which is continuous on all ${\varvec{a}}\in {\mathbb {I}}(D)$. The interval extension of *f* is defined by ${\varvec{f}}: {\mathbb {I}}(D) \rightarrow \mathbb {I}$, ${\varvec{f}}({\varvec{a}}):= \{ f(a) \ |\ a \in {\varvec{a}}\}$. The interval extension of a given elementary function can be calculated e.g. by invoking monotonicity properties.

The *n*-dimensional intervals (also called *boxes*) will also be denoted in boldface, with its indices marked in subscripts: ${\varvec{a}}=({\varvec{a}}_1, {\varvec{a}}_2,\dots ,{\varvec{a}}_n)$, ${\varvec{a}}\in {\mathbb {I}}^n$, and ${\varvec{a}}_i \in \mathbb {I}$ for $i=1,2,\dots ,n$. Moreover, for a given set of *n*-dimensional vectors $D \subseteq {\mathbb {R}}^n$, ${\mathbb {I}}(D)$ will denote the set of *n*-dimensional boxes in *D*. For boxes ${\varvec{a}}, {\varvec{b}}\in {\mathbb {I}}^n,\ \mathrm {hull}({\varvec{a}},{\varvec{b}})$ denotes the rectangular (componentwise) hull of ${\varvec{a}}$ and ${\varvec{b}}$. The arithmetic operators and one-dimensional functions are defined componentwise for boxes, similarly as for real vectors.

The interval extensions of compound real functions are called *interval inclusion functions*. We call ${\varvec{f}}: {\mathbb {I}}(D) \rightarrow \mathbb {I}$ an *inclusion function* of $f: D \subseteq {\mathbb {R}}^n \rightarrow {\mathbb {R}}$, if $f({\varvec{x}}) = \{f(x)\, |\, x \in {\varvec{x}}\} \subseteq {\varvec{f}}({\varvec{x}})$ holds for all ${\varvec{x}}\in {\mathbb {I}}(D)$, where $f({\varvec{x}})$ denotes the range of *f* over ${\varvec{x}}$. One of the possible ways of constructing such interval functions is the so-called *natural interval extension*: in the real-type function expression the variables are replaced by intervals, and the operators and elementary functions are replaced by the corresponding interval ones. Note that usually $f({\varvec{x}}) \subset {\varvec{f}}({\varvec{x}})$ holds for the interval inclusion functions, that is, the interval evaluation *overestimates* the exact range.

For the computer implementation of interval calculations with finite precision floating-point arithmetic, it is essential to control the occurring rounding errors, in order to reach mathematical rigor for the results of calculations (e.g., to guarantee that the above basic inclusion properties hold). This is usually done by the respective interval software packages, using exactly representable floating-point numbers (also called machine numbers) as the bounds of the intervals, and applying directed outward rounding during the calculations.

## An interval branch and bound algorithm

In this section an interval branch and bound method is presented for computing interval enclosures of all global maximizers and the $f^*$ maximum value of the global optimization problem

3 $$\begin{aligned} \max _{z \in {\varvec{z}}} f(z), \end{aligned}$$

where $f: {\mathbb {R}}^n \rightarrow \mathbb {R}$ is a continuous objective function and ${\varvec{z}}\in {\mathbb {I}}^n$ is the search box. The pseudo-code of the method is given in Algorithm 1.

In Algorithm 1 we maintain two sets: ${\mathcal {W}}$ stores the current leaves of the B&B tree, while in ${\mathcal {R}}$ the candidate enclosures of the global maximizers are stored. In both of these sets we store the pairs $({\varvec{u}},{\mathrm{sup}}({\varvec{f}}({\varvec{u}}))$ for the subbox ${\varvec{u}}$, where ${\varvec{f}}$ is an interval inclusion function of *f*. In each iteration cycle (between Step 2 and Step 11 of Algorithm 1), a leaf is chosen and bisected. The leaf selection method is discussed in the next paragraph. The bisection methods used in the current study are very specific to the problem and are detailed in Sect. 7. Then for both ${\varvec{u}}^k$ subboxes we attempt to delete those parts of ${\varvec{u}}^k$ that cannot contain a global optimizer (Step 8). If the remaining part of ${\varvec{u}}^k$ (denoted also by ${\varvec{u}}^k$ in the algorithm) fulfills the termination criterion, we store it in ${\mathcal {R}}$ (Step 10), otherwise we place it in ${\mathcal {W}}$ for further processing (Step 11). The search is completed when ${\mathcal {W}}$ becomes empty.
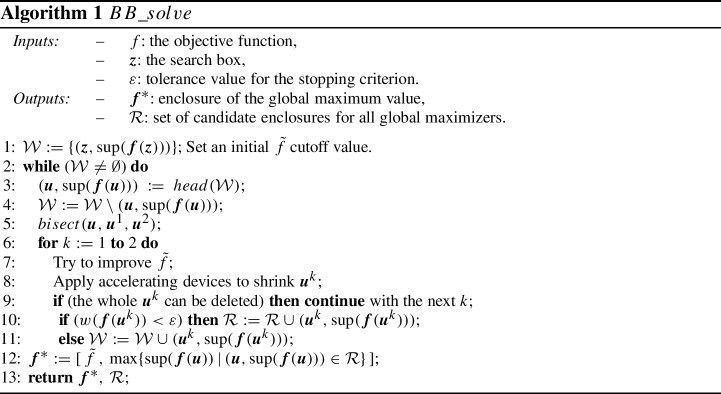


In the current study both ${\mathcal {W}}$ and ${\mathcal {R}}$ have been implemented with the multiset container of the Standard Template Library, storing the elements in decreasing order according to the ${\mathrm{sup}}({\varvec{f}}({\varvec{u}}))$ field. Hence in Step 3, the function *head* returns the leaf with the largest upper bound of the interval inclusion function value.

The algorithmic details discussed up to this point basically followed the techniques used in standard interval B&B frameworks; for further information we therefore refer to the basic textbooks on the subject, e.g., [7, 8]. In contrast, the remaining details of the algorithm, such as the evaluation of ${\varvec{f}}({\varvec{u}})$, the update of ${\tilde{f}}$, and the accelerating devices (Step 8) are specific for the present packing problem and will be discussed below:

*An interval inclusion function of the objective function of the point packing problem.* We use the same inclusion function as in [6], first given in [10]:

### Theorem 1

[10]: Let $({\varvec{x}}, {\varvec{y}}) \subseteq [0,1]^{2n}$, and let ${\varvec{s}}_{ij} = {({\varvec{x}}_{i}-{\varvec{x}}_{j})}^2 + {({\varvec{y}}_{i}-{\varvec{y}}_{j})}^2$ for all $i,j \in \{1, 2, \ldots , n\}$. An inclusion function of $f_n(x,y)$ over the 2*n*-dimensional box $({\varvec{x}},{\varvec{y}})$ is given by

$$\begin{aligned} {\varvec{f}}_n({\varvec{x}},{\varvec{y}}) := [\min _{1 \le i< j \le n} {\mathrm{inf}}({{\varvec{s}}}_{ij}), \min _{1 \le i < j \le n} {\mathrm{sup}}({{\varvec{s}}}_{ij}) ]. \end{aligned}$$

Note that in general the above inclusion function overestimates the exact function range.

*Updating* ${\tilde{f}}$. In Algorithm 1, the value ${\tilde{f}}$ denotes the currently best known guaranteed lower bound for the global maximum, used for eliminating suboptimal boxes (or parts of them). In a general framework, this value is initialized as early as possible, and is updated regularly, e.g., by computing the interval inclusion function value on feasible points.

For practical considerations, we will use the notations $\tilde{f_0}$ and ${\tilde{f}}$ as the lower bounds of the objective functions in (1) and (2), resp. For the present packing problem instances these initial values were determined from the currently best-known packing configurations (see Sect. 7.2 for details). Although a simple updating mechanism was built into the algorithm, the initial values had never been updated, because as we expected the known best configurations have been proven to be the globally optimal ones.

*Accelerating devices.* In general, in Step 8 of Algorithm 1 several tests are performed to delete those parts of ${\varvec{u}}^k$ that cannot contain global maximizer points. In some cases the whole box can be rejected. Using the lessons learned from the predecessor interval algorithms in [6, 10], in the current algorithm we employ only one accelerating test, the *so-called method of active areas*, that is actually the key local method of the whole computer-assisted proof. This method is originated from the first non-interval based computer-aided proofs of the problem class [2, 5, 11].

The method is outlined as follows: Assume we have a validated $\tilde{f_0}$ value. Consider ${\varvec{u}}^k$ in the form of $({\varvec{x}},{\varvec{y}}) \subseteq [0,1]^{2n}$; then for each $i=1,\dots ,n$, the pair $({\varvec{x}}_i,{\varvec{y}}_i) \subseteq [0,1]^2$ is a rectangle in the unit square containing the *i*th point to be placed. These rectangles are called the *initial active regions*.

During the procedure, from each active region $R_i$ we can delete those points that have a distance smaller than $\tilde{f_0}$ to *all* points of another active region $R_j,\ j\ne i$. Once a region is reduced, it can be further used to reduce the ‘neighboring’ regions, thus, the elimination step can be repeated iteratively for all pairs of regions. The procedure ends when either a region becomes empty (which proves that ${\varvec{u}}^k$ is suboptimal, hence, it can be fully eliminated) or a pre-given iteration limit is reached. In the latter case, ${\varvec{u}}^k$ can be updated with the remaining active regions. For a more detailed description and a pseudo-code see [6].

The most crucial part of the algorithm is the representation of the intermediate active areas (i.e., the $R_i$ regions). As pointed out in [6], the set of points of a two-dimensional geometric object having a distance at least $\tilde{f_0}$ from all points of another object may be nonconvex or even non-connected. However, a good approximation of the active (and inactive, i.e., erasable) point sets is essential for the efficient execution of the method. In [2] the initial active regions have been split horizontally and vertically into small rectangular pieces. In [11] a similar approach was used, but using splittings in only one direction. The most effective approximation of the non-interval methods was the one of Nurmela and Östergård [5], that approximated the active regions by polygons.

The predecessor interval method [6] of the present study also used a polygon approach. In that method, the polygons have been represented by a sequence of machine representable points (pairs of machine numbers), and in all elimination steps, reliable calculations have been made to ensure that the result polygon always encloses the one that would be computed by exact calculations. However, that approach resulted in a quite complex algorithm with a tedious proof of correctness, and it led to a large, hard-to-maintain, and relatively slow code. The full description of that method actually required a separate paper [12].

In the present paper we introduce a much simpler reliable polygon representation that saves most of the tedious calculations and case examinations, thus, it results in a simpler proof of correctness and a more efficient program code. The new method will be detailed in the next section.

## An improved method of active areas using interval polygons

In this section, a *convex polygon* $R \subseteq \mathbb {R}^2$ will be defined by the sequence of its vertices $r_1, \dots ,r_m,\ r_i \in \mathbb {R}^2,\ i=1,\dots ,m$, so that the edges of *R* are the line segments $\overline{r_1r_2}, \overline{r_2r_3}, \dots , \overline{r_mr_1}$. When emphasizing the vertices of *R* we will use the notation $R(r_1, \dots ,r_m)$. We consider the cases $m=1$ (i.e., a single point) and $m=2$ (i.e., a line segment) also as polygons. The set of vertices of *R* will be denoted by *V*(*R*), i.e., $V(R) = \{r_1, \dots ,r_m\}$.

The euclidean distance between two points *p* and *q* will be denoted by *d*(*p*, *q*). If *Q* is a set of points, the *maximum* distance between *p* and *Q* will be denoted by *d*(*p*, *Q*), i.e., $d(p,Q) = \max _{q \in Q} d(p,q)$. If *P* and *Q* are sets of points, we will use the notation $d(P,Q) = \max _{p \in P, q \in Q} d(p,q)$.

We begin by introducing an exact version of the active area elimination method, originated from [5]:

### Lemma 1

[5]: If a point *p* is at distance less than $\tilde{f_0}$ from all vertices of a polygon *R*, it is at distance less than $\tilde{f_0}$ from all points of *R*. Formally: $d(p,V(R))< \tilde{f_0} \ \Rightarrow \ d(p,R) < \tilde{f_0}$.

### Theorem 2

[5]: Assume that $p_1,\dots ,p_k$ are distinct points on the boundary of a polygon $R_i$, so that the line segments $\overline{p_{\ell }p_{\ell +1}}$ for $2 \le \ell \le k-2$ are successive edges of $R_i$, and that $\overline{p_1p_2}$ and $\overline{p_{k-1}p_k}$ lay on the edges of $R_i$. Furthermore, assume that $d(p_i,V(R_j)) < \tilde{f_0}$ for $1 \le i \le k$. Then for the polygon $R = R(p_1,p_2,\dots ,p_k)$ we have $d(R,R_j) < \tilde{f_0}$.

**Fig. 1:**
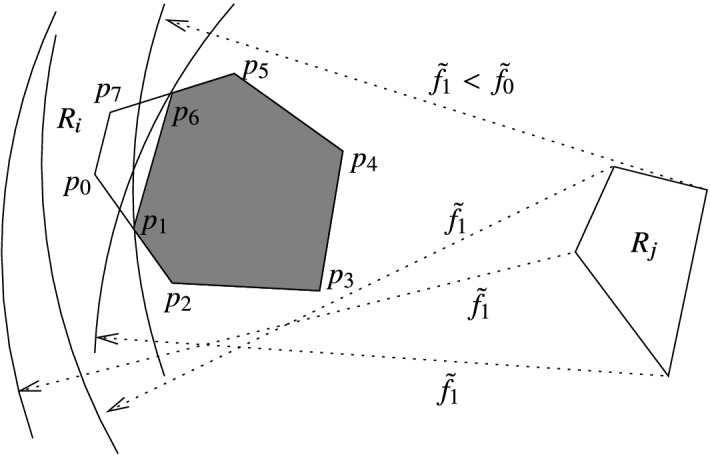
An example of using Theorem 2

Figure 1 illustrates the method based on the above theorem for $k=6$ (indexing the vertices so that they fit to the index settings of the theorem), reducing the polygon $R_i(p_0,\dots ,p_7)$. The theorem can be applied by drawing arcs of radius ${\tilde{f}}_1 < \tilde{f_0}$ from all vertices of $R_j$, and setting that of the intersection point on $\overline{p_0p_2}$ (resp., $\overline{p_5p_7}$) to $p_1$ (resp., $p_6$) which is the ‘closest’ to $p_2$ (resp., to $p_5$). Then all points of the shaded polygon can be eliminated by the active area method, and the polygon $R_i' = R_i'(p_0,p_1,p_6,p_7)$ can be considered in place of $R_i$ as the remaining active region.

Due to the importance of the special intersection points we introduce the following definition:

### Definition 1

Let $\overline{p_0p_2}$ be a line segment and *Q* be a set of points, and assume that $d(p_2,Q) < \tilde{f_0}$. Then a point $p \in \overline{p_0p_2}$ with $d(p,Q) < \tilde{f_0}$ will be called a *reduction point* on $\overline{p_0p_2}$ with respect to *Q*.

Thus, the above theorem says that any pair of reduction points $p_1$ and $p_k$ w.r.t. $V(R_j)$ are suitable to form the remaining active region. Also note that if $p_1$ and $p_k$ are reduction points, then any points on the line segments $\overline{p_1p_2}$ and $\overline{p_{k-1}p_k}$, resp., are also reduction points.

It is important to note that if the polygons $R_i,\ i=1,\dots ,n$ are initialized as convex sets (as in the current study, since we start with rectangles), then they remain convex after each elementary reduction made by Theorem 2. This is because the remaining polygons will always be the intersection of a convex polygon and one of the half planes determined by $\overline{p_1p_k}$.

However, like for many geometric algorithms, the points $p_1$ and $p_k$ cannot be evaluated exactly with finite precision floating point arithmetic. Hence we need a mathematically rigorous version of the above method.

Next, we introduce the interval concepts and notation to develop the alternative interval version of the method in [6, 12].

### Definition 2

A convex interval polygon ${\varvec{R}}$ is defined by the sequence of its vertices ${\varvec{r}}_1, \dots ,{\varvec{r}}_m$, where $m \ge 1$ and ${\varvec{r}}_i \in \mathbb {I}^2,\ i=1, \dots ,m$ are two-dimensional, pairwise disjoint intervals. As a set theoretical definition, ${\varvec{R}}$ is the set of all convex polygons $R = R(r_1, \dots ,r_m), \ i=1,\dots ,m$, where $r_i \in {\varvec{r}}_i,\ i=1, \dots ,m$.

Note that a convex interval polygon given by ${\varvec{r}}_1, \dots ,{\varvec{r}}_m$ may be empty if no convex polygon can be formed from its vertices.

The disjointedness of the interval vertices in the definition is important to get the enclosed polygons easily. This assumption substantially simplifies — in contrast to the predecessor algorithm of [6, 12] — the treated polygon shapes. Note that the disjointedness may fail during the iterative execution of the algorithm, in particular, when the interval vertices are getting so large (due to interval overestimation), that their size is comparable to the polygon itself. However, this actually causes no significant problem for the present method, since we anyway limit the number of iterative area reduction steps, as mentioned above. The disjointedness of the computed interval vertices is very easy to verify, and whenever this criterion fails, we make no area reduction of that interval polygon in the particular reduction step. Furthermore, in the final local refinement phase of the optimality proof we switch to a version that limits the growth of the interval vertices, thus, allows high precision estimates of the final remaining regions (see Sect. 5.2 below for the details).

The distance notation of the exact version can be naturally used to intervals and rectangles the following way:

The maximum euclidean distance between two two-dimensional intervals ${\varvec{p}}$ and ${\varvec{q}}$ is given by $d({\varvec{p}},{\varvec{q}}) = \max _{p \ \in {\varvec{p}}, q \in {\varvec{q}}} d(p,q)$. If *Q* is a set of two-dimensional intervals, the maximum distance between ${\varvec{p}}$ and *Q* is given by $d({\varvec{p}},Q) = \max _{{\varvec{q}}\in Q} d({\varvec{p}},{\varvec{q}})$. If *P* and *Q* are sets of two-dimension intervals, we have $d(P,Q) = \max _{{\varvec{p}}\in P, {\varvec{q}}\in Q} d({\varvec{p}},{\varvec{q}})$.

It is important to note that since we are working with two-dimensional boxes, tight interval enclosures of the above quantities can be computed very fast using interval arithmetic. Let ${\varvec{d}}$ denote the interval inclusion function of *d*. If we obtain, for example, that ${\mathrm{sup}}({\varvec{d}}) < {\tilde{f}}_0$, then we have $d < {\tilde{f}}_0$, that is, we have the mathematical guarantee that all distances within the arguments of *d* are certainly less than ${\tilde{f}}_0$.

**Fig. 2:**
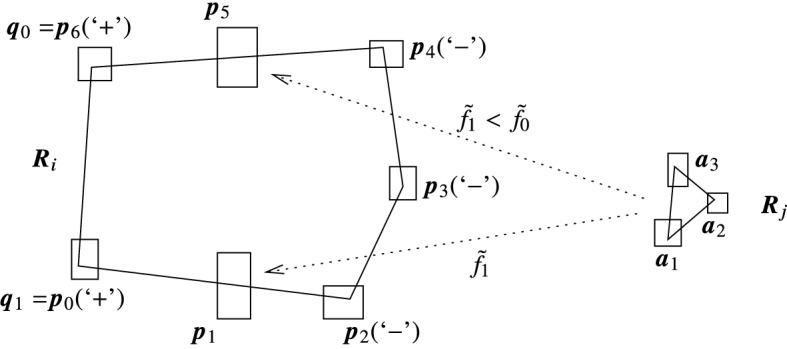
The interval area elimination method

The interval version of the area elimination method is given in Algorithm 2. The method is demonstrated in Fig. 2. In the figure ${\varvec{R}}_i$ has five vertices (marked either with ‘−’ or with ‘$+$’ during the execution of the algorithm), while ${\varvec{R}}_j$ has three. A possible contained polygon is depicted for both of them by solid lines. The remaining polygon ${\varvec{R}}_i'$ (that is an enclosure of an exact remaining polygon, see Theorem 3 below) consists of the vertices ${\varvec{p}}_1,{\varvec{p}}_5,{\varvec{q}}_0,{\varvec{q}}_1$. The case $s=2$ could be visualized by considering the interval line segment with endpoints ${\varvec{p}}_0$ and ${\varvec{p}}_2$ as ${\varvec{R}}_i$.
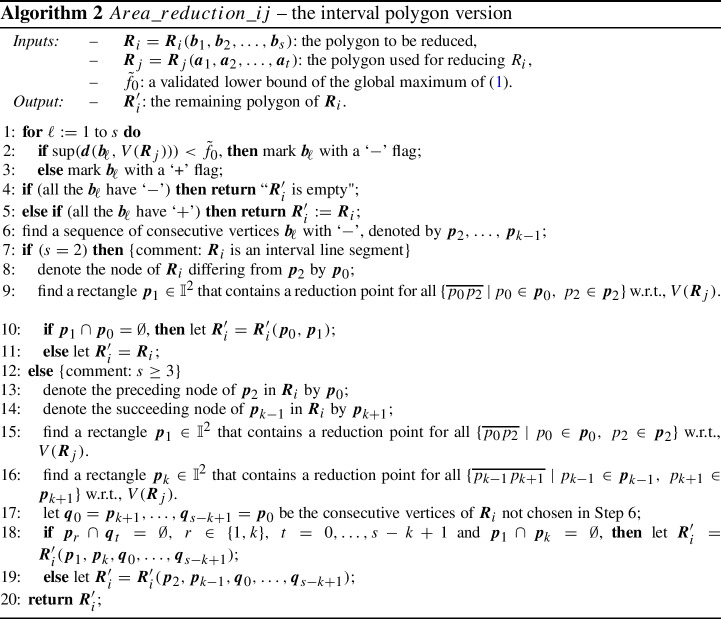


The detailed analysis of the algorithm will be provided at the proof of its correctness below:

### Theorem 3

Algorithm 2 is correct in the sense that for any pair of convex polygons $R_i \in {\varvec{R}}_i, R_j \in {\varvec{R}}_j$, the reduced polygon ${\varvec{R}}_i'$ contains that of the polygon that would be a possible output of the exact area elimination method carried out for $R_i$ and $R_j$.

### Proof

Let $R_i \in {\varvec{R}}_i$, that is, $b_{\ell } \in {\varvec{b}}_{\ell },\ \ell =1,\dots ,s$, and let $R_j \in {\varvec{R}}_j$. Observe that the condition ${\mathrm{sup}}({\varvec{d}}({\varvec{b}}_{\ell },V({\varvec{R}}_j)) < \tilde{f_0}$ in line 2 implies $d(b_{\ell },V(R_j)) < \tilde{f_0}$. Hence if we find that ${\mathrm{sup}}({\varvec{d}}({\varvec{b}}_{\ell },V({\varvec{R}}_j))) < \tilde{f_0}$ holds for all ${\ell }$, then $d(b_{\ell },V(R_j)) < \tilde{f_0}$ holds for all ${\ell }$ as well, so $R_i \in {\varvec{R}}_i$ can be fully eliminated by the exact algorithm. Since $R_i$ and $R_j$ are chosen arbitrarily, in this case we can eliminate ${\varvec{R}}_i$ as a whole in line 4. If all vertices are marked with a ‘$+$’, then we cannot compute reduction points, therefore in line 5 we return ${\varvec{R}}_i' = {\varvec{R}}_i$ with no reduction. Now we continue by analyzing the cases for the different *s* values.

First consider the case $s=1$, that is, ${\varvec{R}}_i = {\varvec{R}}_i({\varvec{b}}_1)$, and $R_i(b_1) \in {\varvec{R}}_i({\varvec{b}}_1)$. By the above discussion, if ${\mathrm{sup}}({\varvec{d}}({\varvec{b}}_1,V({\varvec{R}}_j))) < \tilde{f_0}$, then any $b_1 \in {\varvec{b}}_1$ can be eliminated by the exact algorithm. In this case we eliminate ${\varvec{R}}_i$ (line 4). Otherwise, $b_1 \in {\varvec{b}}_1$ may or may not be eliminated by the exact algorithm, so a possible correct output of the exact algorithm is to keep $b_1$. In this case we keep ${\varvec{R}}_i = {\varvec{R}}_i({\varvec{b}}_1)$ as a whole (line 5).

Next consider $s=2$. If we arrive at line 7, then we have ${\varvec{R}}_i = {\varvec{R}}_i({\varvec{p}}_0,{\varvec{p}}_2)$, where ${\varvec{p}}_0$ is marked with ‘−’ and ${\varvec{p}}_2$ is marked with ‘$+$’. Then in line 9 we compute ${\varvec{p}}_1$, which is by definition the *enclosure* of possible reduction points for all choices $p_0 \in {\varvec{p}}_0,\ p_2 \in {\varvec{p}}_2$. That is, for any $R_i(p_0,p_2) \in {\varvec{R}}_i({\varvec{p}}_0,{\varvec{p}}_2)$ (i.e., $p_0 \in {\varvec{p}}_0,\ p_2 \in {\varvec{p}}_2$), a possible reduction point of the exact algorithm will be contained in ${\varvec{p}}_1$. Hence, a possible outcome of the exact algorithm is to have $R_i'= R_i'(p_0,p_1)$ as the remaining polygon, that will be contained in the output polygon ${\varvec{R}}_i'({\varvec{p}}_0,{\varvec{p}}_1)$ of line 10.

In line 10 we also check whether ${\varvec{p}}_1$ is disjoint from ${\varvec{p}}_0$, so that we are able to form a convex interval polygon. If this is not the case, in line 11 we return the original polygon as a whole, which is also a correct output for the exact algorithm. This completes the proof for $s=2$.

Finally consider $s \ge 3$. The line of thought is similar to $s=2$, using two reduction points: if we are in line 12, then we have the consecutive vertices ${\varvec{p}}_2,\dots ,$ ${\varvec{p}}_{k-1}$ marked with ‘−’. In lines 15 and 16 we compute the enclosures ${\varvec{p}}_1$ and ${\varvec{p}}_k$ of possible reduction points $p_1$ and $p_k$ for all choices of $p_0 \in {\varvec{p}}_0,\ p_2 \in {\varvec{p}}_2$ and $p_{k-1} \in {\varvec{p}}_{k-1},\ p_{k+1} \in {\varvec{p}}_{k+1}$, resp. That is, for any $R_i \in {\varvec{R}}_i$ a possible outcome of the exact algorithm is to have $R_i' = R_i'(p_1,p_k,d_0 \in {\varvec{q}}_0,\dots ,d_{s-k+1} \in {\varvec{q}}_{s-k+1})$ as the remaining polygon.

If the disjointedness property of the rectangles ${\varvec{p}}_1,{\varvec{p}}_k,{\varvec{q}}_0,\dots ,{\varvec{q}}_{s-k+1}$ holds in line 18, then we have ${\varvec{R}}_i' = {\varvec{R}}_i'({\varvec{p}}_1,{\varvec{p}}_k,{\varvec{q}}_0,\dots ,{\varvec{q}}_{s-k+1})$ and thus $R_i' \in {\varvec{R}}_i'$. If the disjointedness fails, we can legally consider $p_2$ and $p_{k-1}$ instead $p_1$ and $p_{k}$ as reduction points of the exact algorithm. The exact algorithm will then result in $R_i' = R_i'(p_2,p_{k-1},d_0 \in {\varvec{q}}_0,\dots ,d_{s-k+1} \in {\varvec{q}}_{s-k+1})$, which is again contained in the ${\varvec{R}}_i'$ polygon constructed in line 19. This concludes the proof.$\square$

Note that in Algorithm 2, ${\varvec{p}}_0 = {\varvec{p}}_{k+1}$ may hold after Steps 13 and 14; in this case we construct ${\varvec{R}}_i'$ without duplicating ${\varvec{q}}_0 = {\varvec{p}}_{k+1}$ and ${\varvec{q}}_{s-k+1} = {\varvec{p}}_0$ in the result polygon.

### Computing reduction rectangles

The only remaining part of Algorithm 2 to discuss is the construction of the enclosure rectangles in Steps 9, 15, and 16. Likewise to the exact version, we will call these enclosures *reduction rectangles* on the respective interval line segment with respect to the set of reducing points.

In the current packing algorithm the computation of the reduction rectangles is implemented in two flavours. In the first method we consider $\overline{{\varvec{p}}_0{\varvec{p}}_2}$ as a simple interval extension of a line segment, where both endpoints are rectangles instead of points. The computation of a reduction rectangle w.r.t. a single vertex ${\varvec{q}}$ is based on solving the interval system describing the intersection of $\overline{{\varvec{p}}_0{\varvec{p}}_2}$ (with ${\mathrm{sup}}({\varvec{d}}({\varvec{p}}_2,{\varvec{q}})) < \tilde{f_0}$) with a set of circles of radius $\tilde{f_1} < \tilde{f_0}$ centered at any $q \in {\varvec{q}}$. The solution procedure is essentially identical to the method described in [12] for the previous implementation, the only difference is that in [12] the endpoints of the line segment and the circle center were treated as thin intervals, while in the present version they are most often thick intervals. However, the interval calculations go exactly the same way, so in the present paper we skip its details. The method is safeguarded in such a way that in case of any computational errors (e.g. an interval-valued discriminant containing negative numbers due to overestimation) the procedure returns ${\varvec{p}}_2$ as the reduction rectangle, which is, by definition, always a proper choice.

In order to compute a reduction rectangle w.r.t. a set of interval vertices (such as $V({\varvec{R}}_j)$ as in Algorithm 2) we first compute the reduction intervals w.r.t. each vertex one by one, and then create the final ${\varvec{p}}_1$ w.r.t. $V({\varvec{R}}_j)$ by properly merging (some of the) the individual reduction rectangles. This merging procedure is also described in [12].

### An improved method for computing reduction rectangles

The above first method of computing reduction rectangles w.r.t. $V({\varvec{R}}_j)$ has been found to be very fast and efficient in the current implementation, however, it has one drawback. Since the endpoints of the input line segments and the centers of the reducing sets are all intervals (vertices of interval polygons), an excessive blowup of the resulting reduction rectangles (the new polygon vertices) occur after a few iterations, due to interval overestimation. Our experience of solving the packing problems revealed that *the initial phases of the algorithm profit from this method, since it eliminates most of the suboptimal search space even before the blowup takes its effect. The final refinement phase of the optimality proof requires, however, a version with smaller overestimation, in order to produce high precision enclosures for the global maximizers*.

**Fig. 3:**
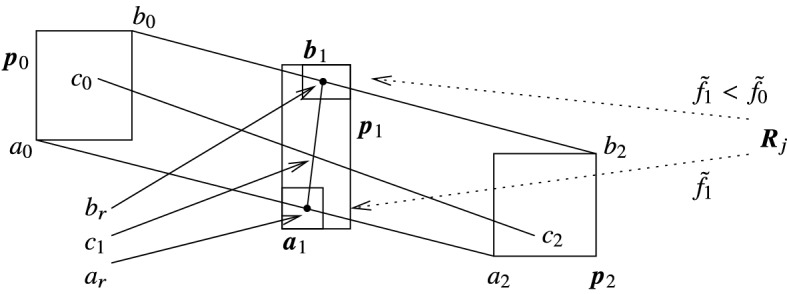
The improved method for computing reduction rectangles

The second, high precision method is based on the idea that instead of computing the intersection of circles with all possible line segments in an interval line segment (a stripe), the reduction rectangle can be computed by intersecting only with the extremal line segments of the stripe. The theorem below shows how this is carried out (using the notation of Algorithm 2). The application of the theorem is shown in Fig. 3.

#### Theorem 4

Let ${\varvec{p}}_0$ and ${\varvec{p}}_2$ be interval vertices and assume that Algorithm 2 marks ${\varvec{p}}_2$ with ‘−’ (i.e., ${\mathrm{sup}}({\varvec{d}}({\varvec{p}}_2,V({\varvec{R}}_j))) < \tilde{f_0}$) and ${\varvec{p}}_0$ with ‘$+$’, resp. Let $\mathrm {conv}({\varvec{p}}_0,{\varvec{p}}_2)$ denote the convex hull of ${\varvec{p}}_0$ and ${\varvec{p}}_2$, and let $\overline{a_0a_2}$ and $\overline{b_0b_2}$ be the line segments on the boundary of $\mathrm {conv}({\varvec{p}}_0,{\varvec{p}}_2)$ that join ${\varvec{p}}_0$ and ${\varvec{p}}_2$. Assume that these two line segments do not lie on the same line. Compute the reduction rectangles on $\overline{a_0a_2}$ and $\overline{b_0b_2}$ w.r.t. $V({\varvec{R}}_j)$, denoted by ${\varvec{a}}_1$ and ${\varvec{b}}_1$, resp. Then ${\varvec{p}}_1 := \mathrm {hull}({\varvec{a}}_1,{\varvec{b}}_1)$ is a reduction rectangle for $\overline{{\varvec{p}}_0{\varvec{p}}_2}$ w.r.t. $V({\varvec{R}}_j)$.

#### Proof

Assume the contrary of the statement, that is, that ${\varvec{p}}_1$ is not a proper reduction rectangle. Then there exists a line segment $\overline{c_0c_2} \in \overline{{\varvec{p}}_0{\varvec{p}}_2}$ for which $\overline{c_0c_2} \cap {\varvec{p}}_1$ contains no reduction point w.r.t. $V({\varvec{R}}_j)$. That is, $\forall p \in \overline{c_0c_2} \cap {\varvec{p}}_1$ we have $d(p,V({\varvec{R}}_j)) \ge \tilde{f_0}$. This implies the existence of a set $Q \subseteq V({\varvec{R}}_j)$ such that $d(p,Q) \ge \tilde{f_0}\ \forall p$, i.e., $\overline{c_0c_2} \cap {\varvec{p}}_1$ contains no reduction point w.r.t. *Q*. Let $a_r \in {\varvec{a}}_1$ denote a reduction point on $\overline{a_0a_2}$ w.r.t. *Q*, and let $b_r \in {\varvec{b}}_1$ denote a reduction point on $\overline{b_0b_2}$ w.r.t. *Q*. (Such points exist, because ${\varvec{a}}_1$ and ${\varvec{b}}_1$ are reduction rectangles w.r.t. $V({\varvec{R}}_j)$, and thus, also w.r.t. *Q*.) Then we have $d(a_r,Q)< \tilde{f_0},\ d(b_r,Q) < \tilde{f_0}$, that is, $d(a_r,q)< \tilde{f_0},\ d(b_r,q) < \tilde{f_0},\ \forall q \in Q$. Let $c_1 = \overline{c_0c_2} \cap \overline{a_rb_r} \in {\varvec{p}}_1$. Then, by Lemma 1 we have $d(c_1,q) < \tilde{f_0},\ \forall q \in Q$, that is, $d(c_1,Q) < \tilde{f_0}$. This means that $c_1$ is a reduction point on $\overline{c_0c_2}$ w.r.t. *Q*. But this contradicts the previous assumption that $\overline{c_0c_2} \cap {\varvec{p}}_1$ contains no reduction point on $\overline{c_0c_2}$ w.r.t. *Q*.$\square$

In this second version we thus calculate reduction rectangles for two real line segments (i.e., with thin intervals as their endpoints), which significantly reduce the overestimation. Then the reduction rectangle for the whole $\overline{{\varvec{p}}_0{\varvec{p}}_2}$ is constructed by taking their rectangular hull. Note that computing the required convex hull of two rectangles and determining $\overline{a_0a_2}$ and $\overline{b_0b_2}$ can be carried out fast, thus the time requirement of executing this improved method is roughly twice of the first one. Also note that if $\overline{a_0a_2}$ and $\overline{b_0b_2}$ lie on the same line, then we necessarily have thin components in ${\varvec{p}}_0$ and ${\varvec{p}}_2$, and we can compute the reduction rectangle by one application of the first method.

In addition to the easier implementation, proof of correctness, and improved efficiency, the reduction algorithm presented in this section has one more important advantage over the predecessor method of [6, 12]. Namely, in the current implementation the complexity of the data structure, i.e., the number of interval vertices, can be kept better under control during the execution of the algorithm. In particular, the present method so closely resembles that of the exact area reduction method, that in most cases the number of interval vertices of each polygon was found to be close to the number of possibly neighboring (i.e., reducing) other polygons of the packing configuration plus the number of sides of the unit square on which the polygon was possibly located.

## A global elimination procedure

In the previous two sections we introduced the branch–and–bound framework designed for point packing problems, with the method of active areas as its key element. The latter method works well for cases where the locations of the points of the packing are at least approximately known. It is clear that if we start the global search from the whole initial box $[0,1]^{2n}$, branching alone will not be sufficient to reach this state in a reasonable amount of steps, due to the problem dimensionality and the difficulties caused by the permutation and symmetry of the points to be packed. Thus, special methods are needed to tackle the initial phase of the global search. The most important such method, used already in [2, 5, 6, 10, 11], is called tiling:

Assume that a lower bound $\tilde{f_0}$ for the maximum value of the point packing problem (1) is given. Split the unit square into regions (tiles), so that the distance between any two points in each tile is less than $\tilde{f_0}$ (or equivalently, that the squared distance between any two points in each tile is less than ${\tilde{f}}$ for (2)). Then for each packing configuration attaining objective function value greater than or equal to $\tilde{f_0}$ each tile can contain at most one point of the packing. The packing problem can be solved to global optimality by running a search procedure on all possible tile combinations.

Due to the rectangular branch-and-bound framework, in our study we prefer a rectangular splitting of the square. Furthermore, we require a regular splitting in order to be able to exploit symmetry and the tile pattern methods (introduced later in this section). If we split the unit square into $k \times l$ rectangles (in a regular way), the minimal number of initial tile combinations is given by

$$\begin{aligned}\min \left\{ \left( {}^{k\cdot l}_{\,\,n}\right) \ | \ k,l \ge 1 \ \mathrm{integers}, \ (1/k^2 + 1/l^2)^{1/2} < \tilde{f_0}\right\} .\end{aligned}$$

In the studies prior to [6], all tile combinations have been eliminated one after the other. Nevertheless, the growth of the number of tile combinations obstructs the solution of the problem instances $n \ge 28$ with this strategy in acceptable time. For $n=28,29,30$ at least a $6\times 7$ tiling is needed, which gives $$\left( {\begin{array}{c}42\\ 28\end{array}}\right) \approx 5.29\cdot 10^{10}$$, $$\left( {\begin{array}{c}42\\ 29\end{array}}\right) \approx 2.55\cdot 10^{10}$$, and $$\left( {\begin{array}{c}42\\ 30\end{array}}\right) \approx 1.11\cdot 10^{10}$$ tile combinations, resp., to be checked.

One of the key ideas of the predecessor method [6] was the observation that instead of processing all tile combinations consisting of *n* tiles, we can first investigate subsets of the full tile combinations, in order to discover patterns of tile sets that cannot contain components of an optimal solution. Then the higher dimensional subproblems containing any of these patterns can be discarded. With the resulting method we were able to solve those three instances in about 53, 50, and 21 CPU hours, with an at that time decent computer architecture.

For the next problems $n=31,32,33$, an $6\times 8$ tiling gives the smallest number of combinations, resulting in $$\left( {\begin{array}{c}48\\ 31\end{array}}\right) \approx 4.24\cdot 10^{12}$$, $$\left( {\begin{array}{c}48\\ 32\end{array}}\right) \approx 2.25\cdot 10^{12}$$, and $$\left( {\begin{array}{c}48\\ 33\end{array}}\right) \approx 1.09\cdot 10^{12}$$ cases, an increase of about 100 times as compared to the previous three cases for each pair of problem instances. Clearly, to reach again a reasonable solution time (and to lay the foundations of solving further instances) we need an improvement of the global phase methods of [6] as well.

Let us denote $P(m,{\varvec{x}}_1,\dots ,{\varvec{x}}_m,{\varvec{y}}_1,\dots ,{\varvec{y}}_m)$ a point packing problem instance where *m* is the number of points to be packed, $({\varvec{x}}_i,{\varvec{y}}_i) \in \mathbb {I},\ i=1,\dots m$ are the components of the starting box (i.e., the rectangle $({\varvec{x}}_i,{\varvec{y}}_i)$ is the location of the *i*th point), and the objective function is given by (2). The theorem and its corollary below from [6] demonstrate how to apply a result achieved on a 2*m*-dimensional packing problem for a higher dimensional problem with 2*k* dimensions, $k\ge m\ge 2$.

### Theorem 5

[6] Let $k \ge m \ge 2$ be integers and let

$$\begin{aligned}&P_m = P(m,{\varvec{z}}_1,\dots ,{\varvec{z}}_m,{\varvec{w}}_1,\dots ,{\varvec{w}}_m) = P(m,({\varvec{z}},{\varvec{w}})), \ \mathrm{and} \\&P_k = P(k, {\varvec{x}}_1,\dots ,{\varvec{x}}_k, {\varvec{y}}_1,\dots ,{\varvec{y}}_k) = P(k,({\varvec{x}},{\varvec{y}})) \end{aligned}$$

be point packing problem instances $(\!{\varvec{x}}_i,{\varvec{y}}_i,{\varvec{z}}_i,{\varvec{w}}_i \in \mathbb {I}, \ {\varvec{x}}_i,{\varvec{y}}_i,{\varvec{z}}_i,{\varvec{w}}_i \subseteq [0,1])$. Run Algorithm 1 on $P_m$ using a hypothetical ${\tilde{f}}$ cutoff value in the accelerating devices and skipping Step 7 (the update of ${\tilde{f}}$), and stop after an arbitrary, preset number of iteration steps. Denote $({\varvec{z}}_1',\dots ,{\varvec{z}}_m',{\varvec{w}}_1',\dots ,$ ${\varvec{w}}_m') := ({\varvec{z}}',{\varvec{w}}')$ the componentwise hull of all elements placed on ${\mathcal {W}}$ and on ${\mathcal {R}}$. Assume that there exists an invertible, distance-preserving geometrical transformation $\varphi$ with $\varphi ({\varvec{z}}_i) = {\varvec{x}}_i$ and $\varphi ({\varvec{w}}_i) = {\varvec{y}}_i,\ \forall i=1,\dots ,m$. Then for each point packing $(x,y) \in {\mathbb {R}}^{2k}$ satisfying $(x,y) \in ({\varvec{x}},{\varvec{y}})$ and $f_k(x,y) \ge {\tilde{f}}$, the statement

$$\begin{aligned}&(x,y) \in \,(\varphi ({\varvec{z}}_1'),\dots ,\varphi ({\varvec{z}}_m'),{\varvec{x}}_{m+1},\dots ,{\varvec{x}}_k, \\&\quad \, \varphi ({\varvec{w}}_1'),\dots ,\varphi ({\varvec{w}}_m'),{\varvec{y}}_{m+1},\dots ,{\varvec{y}}_k) := ({\varvec{x}}',{\varvec{y}}') \end{aligned}$$

also holds.

Informally Theorem 5 states the following: assume that we are able to reduce some search regions on a tile set $S'$. When processing a higher dimensional subproblem (using the same cutoff value) on a tile set *S* containing the image of $S'$, it is enough to consider *the image of those of the remaining regions of* $S'$ for the particular components of *S*.

### Corollary 1

[6] Let $P_m, P_k$, ${\tilde{f}}$, and $\varphi$ be as in Theorem 5. Let $\varphi$ be the identity transformation and assume that Algorithm 1 stops with ${\mathcal {W}} = \emptyset$ and ${\mathcal {R}} = \emptyset$, i.e. the whole search region $({\varvec{z}},{\varvec{w}}) = ({\varvec{z}}_1,\dots ,{\varvec{z}}_m,{\varvec{w}}_1,\dots ,{\varvec{w}}_m) = ({\varvec{x}}_1,\dots ,{\varvec{x}}_m,$ ${\varvec{y}}_1,\dots ,{\varvec{y}}_m)$ is eliminated by the accelerating devices using ${\tilde{f}}$. Then $({\varvec{x}},{\varvec{y}})$ does not contain any $(x,y)\in {\mathbb {R}}^{2k}$ vectors for which $f_k(x,y) \ge {\tilde{f}}$ holds.

Corollary 1 states that if it is proved that $S'$ cannot contain point packings attaining at least ${\tilde{f}}$ function value, then all higher dimensional problems with the tile set *S*, $S'\subseteq S$ can be eliminated at once (when using the same ${\tilde{f}}$).

In the present study the global search phase is improved by using the two additional theorems below:

### Theorem 6

Let $k \ge 2$ and let

$$\begin{aligned} P_k = P(k, {\varvec{x}}_1,\dots ,{\varvec{x}}_k, {\varvec{y}}_1,\dots ,{\varvec{y}}_k) = P(k,({\varvec{x}},{\varvec{y}})) \end{aligned}$$

be a point packing problem instance $(\!{\varvec{x}}_i,{\varvec{y}}_i \in \mathbb {I}, \ {\varvec{x}}_i,{\varvec{y}}_i \subseteq [0,1])$. Run Algorithm 1 on $P_k$ using a hypothetical ${\tilde{f}}_1$ cutoff value in the accelerating devices and skipping Step 7 (the update of ${\tilde{f}}_1$), and stop after an arbitrary, preset number of iteration steps. Denote $({\varvec{x}}_1',\dots ,{\varvec{x}}_k',{\varvec{y}}_1',\dots {\varvec{y}}_k') := ({\varvec{x}}',{\varvec{y}}')$ the componentwise hull of all elements placed on ${\mathcal {W}}$ and on ${\mathcal {R}}$ and let ${\tilde{f}}_2 > {\tilde{f}}_1$. Then for any point packing $(x,y) \in ({\varvec{x}},{\varvec{y}})$ with $f_k(x,y) \ge {\tilde{f}}_2$ we have $(x,y) \in ({\varvec{x}}',{\varvec{y}}')$.

### Proof

Consider a point packing (*x*, *y*) with $(x,y) \in ({\varvec{x}},{\varvec{y}})$ and $f_k(x,y) \ge {\tilde{f}}_2$ and assume that $(x,y) \not \in ({\varvec{x}}',{\varvec{y}}')$. Then (*x*, *y*) is discarded by Algorithm 1 using ${\tilde{f}}_1$. This implies that $f_k(x,y) < {\tilde{f}}_1$, which together with ${\tilde{f}}_2 > {\tilde{f}}_1$ gives $f_k(x,y) < {\tilde{f}}_2$, a contradiction.$\square$

### Remark 1

The importance of Theorem 6 is that if some search regions on a tile set *S* can be eliminated by a ${\tilde{f}}_1$ cutoff value, then it is sufficient to consider only the reduced regions (on the same tile set) when using a larger ${\tilde{f}}_2$ cutoff value. Note that the cutoff values are computed from the best-known optimal packing solutions, and we have ${\tilde{f}}_{31}> {\tilde{f}}_{32} > {\tilde{f}}_{33}$, see Sect. 7.2. Thus for our present problem instances the *remaining regions computed on the subproblems of* $n=33$ *can be used for the solution process for* $n=32$ *and* $n=31$, *and similarly, the remaining regions computed for* $n=32$ *can be used when solving the case* $n=31$. That is, as a completely new idea, we solve the largest of the considered problem instances first (which anyway consists of the smallest number of tile combinations), and in this way avoid many of the repeated search space reductions for the smaller problem instances.

Another new theorem that helps to improve the global search phase is based on the already known optimal solution for 10 circles. It gives an important property of the tile patterns to be considered.

### Theorem 7

No optimal packing of $n=31,32,33$ points, resp., can contain 10 or more points in a region of size $0.5 \times 0.5$ in the unit square.

### Proof

Assume that there exists an optimal arrangement of $n=31$ (resp., 32, 33) points in the unit square, for which a region of size $0.5 \times 0.5$ contains 10 points. Let the smallest distance between the pairs of points in this arrangement be denoted by $f_{31}$ (resp., $f_{32},f_{33}$). From the best known packing values of $n=31$ (resp., 32, 33), see [13], we have $f_{31} > 0.2175$ (resp., $f_{32} > 0.2131$ and $f_{33} > 0.2113$). Enlarging the mentioned region to the size of a unit square, we get an arrangement of 10 points with the smallest pairwise distance between them being at least $2f_{31} > 0.435$ (resp., $2f_{32} > 0.4262$ and $2f_{33} > 0.4226$). But this contradicts the fact that the known optimum of the problem of packing 10 points is $f^*_{10} < 0.4213$.$\square$

**Fig. 4:**
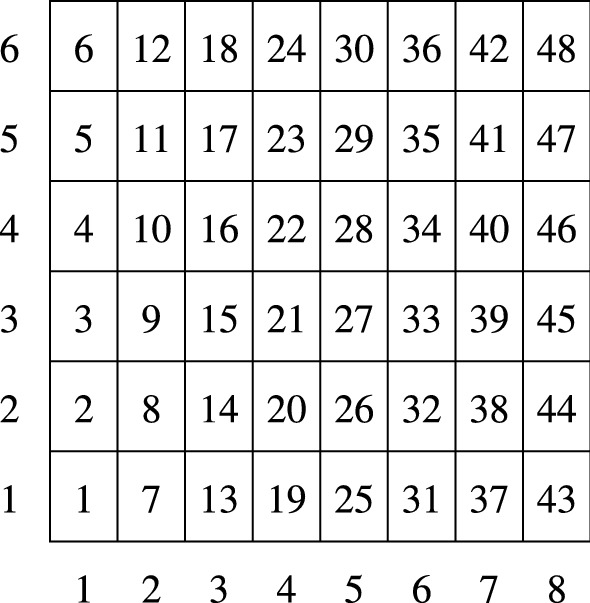
Row, column, and tile numbering for $n=31,32,33$

To achieve mathematical correctness during the application of the symmetry transformations, it is essential that the tiles have identical size even in their computer representation. Therefore, for the $6\times 8$ tiling we enlarge the unit square to $[0,24]^2$ so that each tile has integer coordinates. (Of course, we also increase the used ${\tilde{f}}$ values accordingly for the B&B search.) Figure 4 shows the row, column, and tile numbering used in the present study.

The method of [6] was based on the following strategy (on a $7\times 6$ rectangular tiling): in phase 1, we run the B&B algorithm on the tile combinations of size $7\times 3$ (filtering cases out by using the symmetry group of a rectangle), and stored the remaining combinations together with the rectangular enclosure of each of their remaining tile regions. In phase 2, we computed the possible combinations of size $7\times 4$ (again together with their remaining regions) by adding one column to the results of phase 1 (after extracting symmetric cases when needed). In phase 3, we joined the remaining combinations from phase 2 on columns 1 to 4 and on columns 3 to 6, by checking feasible tile patterns and their active regions on the joint columns 3 and 4. This led us to the remaining regions on the whole square with *n* active regions, one from each tile. The proof process had been completed with two local refinement phases. Although leading to a successful solution, both phases 2 and 3 required quite complicated algorithms, fully detailed in [6].

The goal of the present study is to improve this strategy in such a way that we use simpler algorithms and improve the tile combination reduction techniques by employing Theorem 6 and Theorem 7 and using more advanced data structures for storing tile combinations and their remaining areas. As we will see later, it is enough to use only two phases for the global part: in phase 1, we compute tile combinations on the half square (size $6\times 4$), then in phase 2 we merge them into the full square. At the same time we can keep the number of intermediate combinations of phase 1 to a manageable size (around one million), so that they all fit into memory for a fast execution of the second phase. Our new global strategy will be introduced in detail in the next section.

## Optimality for $n=31,\ 32$, and 33

### Hardware and software environment

The optimization procedure was carried out on a laptop computer with an Intel T2080 1.73 GHz CPU and 2 Gbytes RAM, under the Linux operating system. (The mentioned processor has two physical cores, however, we run the whole process sequentially, so only one core was used at a time.) The algorithms have been implemented in C++, using the C–XSC interval arithmetic library [14].

### Guaranteed lower bounds for the maximum

The currently known best packings have been found by R.L. Graham and B.D. Lubachevsky for $n=31$ and $n=33$ [15], and by D.W. Boll et al. for $n=32$ [16]. In [13] the maximum values and the maximizers are given with the precision of 30 digits. The ${\tilde{f}}_0$ lower bounds have been obtained from the given optimizers, cut to 16 digits after the decimal dot:

$$\begin{aligned}&{\tilde{f}}_{0,31} = 0.2175472916191244, \\&{\tilde{f}}_{0,32} = 0.2131745625898765, \\&{\tilde{f}}_{0,33} = 0.2113283841432631. \end{aligned}$$

Note that one needs to verify that these numbers (more precisely, the *double precision floating point numbers created from these decimal constants*) are indeed lower bounds of the problem. This check has been done by variable precision calculations with GNU Octave, using the above mentioned high precision coordinates. The actual lower bounds used during the calculations (recall that we optimize on $[0,24]^2$ for squared distances) have been constructed by computing $(24 \cdot {\tilde{f}}_{0,n})^2$ with interval arithmetic and taking the infimum of the result.

### Optimality proof for $n=33$

During the optimality proof we work on sets of tile combinations, where each combination is represented by $k < n$ rectangles. Each of these rectangles is initialized with the bounds of the respective full tile and during the process it contains the rectangular enclosure of the remaining region of that tile (after, e.g., running Algorithm 1 or using tile pattern techniques based on Theorems 5–7). In particular, $S_4^k$ will denote the sets of tile combinations where *k* tiles are taken from columns 1 to 4 (i.e., the left half) of the square. Furthermore, $S_8^{\ell ,r}$ with $\ell +r=n$ will denote sets where $\ell$ tile regions are considered from the left half and *r* regions are considered from the right half of the square. Finally, $S_8^n$ denotes the set of combinations where *n* tile regions are taken from the full square. (In the notation we do not differentiate whether these sets are the initial ones with full tiles or they are ones with reduced regions. During the discussion the actual state of these sets will always be made clear.)

The tile positions of the tile combinations were represented by the Standard Template Library (STL) bitset of length 48. This allows fast manipulation of the tile combinations, since symmetry transformations and shifting can be carried out very efficiently on this data structure. Furthermore, ordering relations can also be easily defined on bitsets (e.g., by a lexicographical ordering on all or parts of the bits), thus, fast (logarithmic) searches can be performed on them when looking for a given tile combination (or a combination with a given subset). It was found that the use of this data structure also contributed to the performance improvement as compared to [6] (where simple binary strings had been used for representing tile positions).

Below we detail the procedure for $n=33$ only, since it is running essentially the same way for all three problem instances. The differences arising for $n=32$ and $n=31$ (e.g., the use of Theorem 6) will be discussed in the next subsection. The whole process consists of three phases: in phase 1 tile combinations in $S_4^k$ are processed for the required *k* values. In phase 2 the sets $S_8^{\ell ,r}$, built from the remaining combinations of phase 1, are processed. These two phases consist of the global part of the search, devoted to reduce the tile combinations in $S_8^n$. In the last, local phase, the (small number of) remaining combinations in $S_8^n$ are processed to provide high precision enclosures for all global maximizers.

#### Phase 1

As mentioned above, we start by processing tile combinations in $S_4^k$. For $n=33$ Theorem 7 implies that there is no need to deal with $k \ge 19$ (since in this case a quarter of the square would contain at least 10 tiles, which is impossible for tile combinations containing a global maximizer). Thus, we start with $k=18$. Since at this point there is no previous information about the possible tile locations, we generate the elements of $S_4^{18}$ consisting of full tiles. The resulting $$\left( {\begin{array}{c}24\\ 18\end{array}}\right) $$ combinations are first filtered by using the symmetry group of the rectangle. In practice this means that for each combination we consider its bitset representation, compute the bitsets of the transformed combinations (horizontal and vertical reflection, and rotation around the midpoint), and keep the original combination for further processing only if it gives the smallest bitset among the four. Otherwise the combination is filtered out. Note that since the tiles are rectangular, we are able to use only the symmetries of the rectangle. If the tile combination survives the symmetry filtering, we perform tile pattern filtering on it, using Theorem 7: if it is found that the combination contains at least 10 tiles in any $3\times 4$ tile region of the half square, then the combination is filtered out.

After these two filtering procedures, we proceed by running Algorithm 1 on the combination, to reduce its active regions within each tile. In phase 1, the following settings of the algorithm are used:

1. The maximum number of iterations of the algorithm is set to 20. Note that in this initial phase there is no need to run the algorithm long (a lesson learned from [6]). The goal here is to make some initial area reductions and to eliminate tile combinations that are easily found to be suboptimal.
2. The direction along which the current box is subdivided is determined the following way: First search for the rectangle $({\varvec{x}}_i,{\varvec{y}}_i)$ with the largest area, and bisect this rectangle perpendicular to its larger side. The goal of this subdivision strategy is to apply branching on components for which the method of active areas worked the least efficiently.
3. The reduction rectangles in Algorithm 2 are computed by the method of Sect. 5.1 (the faster one with larger overestimation). This is because in this phase the primary goal is to eliminate combinations fast and it is less important to go for high precision results.
4. The stopping tolerance $\varepsilon$ used in Step 10 of Algorithm 1 is set to $10^{-12}$ (also in the later phases). However, in this phase the termination of the algorithm is mainly controlled by the maximum number of iterations.

If the algorithm stops with no subboxes left, the tile combination cannot be a subset of an optimal combination (see Corollary 1). Otherwise, the tile combination is stored together with the rectangular enclosures of its remaining tile regions for the next phase.

Table 1 contains the computational details of the steps of the optimality proof for $n=33$, grouped by the successive phases. The table is organized the following way: the first column is the notation of the particular set of tile combinations. The second column, marked by $|S|_{th}$, contains, as a reference, the (approximate) theoretical size of this set with no elimination. That is, the exact values in this column are $$\left( {\begin{array}{c}24\\ k\end{array}}\right) $$ for $S_4^k$, $$\left( {\begin{array}{c}24\\ \ell \end{array}}\right) \cdot \left( {\begin{array}{c}24\\ r\end{array}}\right) $$ for $S_8^{\ell ,r}$, and $$\left( {\begin{array}{c}48\\ n\end{array}}\right) $$ for $S_8^n$. The third column, marked by $|S|_{filt}$, contains the size of this set of tile combinations *after performing symmetry filtering, tile pattern filtering (and in phase 2, tile region filtering)*. This is the size of the set that is passed one by one to the $B \& B$ algorithm. The fourth column, marked by $|S|_{red}$ contains the size of the set of remaining tile combinations *after running the B&B algorithm*. The last column contains the overall running time of the B&B method on this set of tile combinations. (Note that the tile generating, filtering, and other auxiliary methods took only about half minute *for the whole optimality proof altogether*, hence their running times are not displayed in the table.)

**Table 1:** Computational details of solving the packing problem of $n=33$ points

*S*	$|S|_{th}$ (approx.)	$|S|_{filt}$	$|S|_{red}$	CPUt (min.)
*Phase 1*				
$S_4^{18}$	$1.34\times 10^5$	6 672	5	0.5
$S_4^{15}$	$1.31\times 10^6$	274 384	153 593	235.3
$S_4^{16}$	$7.35\times 10^5$	39 108	37 825	57.2
$S_4^{17}$	$3.46\times 10^5$	3 152	2 935	4.8
*Phase 2*				
$S_8^{18,15}$	$1.76\times 10^{11}$	87 372	45	3.6
$S_8^{17,16}$	$2.55\times 10^{11}$	15 096 352	346	446.1
*Phase 3*				
$S_8^{33}$	$1.09\times 10^{12}$	391	4	11.3
$S_8^{33}$	$1.09\times 10^{12}$	4	4	14.0
$\sum$	–	15 507 435	194 757	772.8

In the example of processing $S_4^{18}$, the first line of the table is thus interpreted as follows: the total number of combinations in this set is $$\left( {\begin{array}{c}24\\ 18\end{array}}\right) \approx 1.34\cdot 10^5$$. After filtering out by symmetry and by the maximum number of tiles in a quarter, we obtained 6 672 combinations. Algorithm 1 reduced the number of tile combinations in this set to 5, with a running time of 0.5 minute. The subsequent lines of the table are interpreted the same way.

After processing $S_4^{18}$, we proceed the same way with its (to be) complement tile combinations on the whole square, that is, with $S_4^{15}$. Next, the set $S_4^{16}$ is processed. In this step we employ a technique (that is also a novelty of the present study) we call *incremental tile generation*. This is based on the observation that a good portion of the area elimination procedures on 16 full tiles on the half square would be a repeated work of what has been already done for 15 tiles. More precisely, from Theorem 5 it is enough to consider the remaining combinations (together with their remaining tile regions) from the processed set of $S_4^{15}$, and extend these with one more full tile. So for $S_4^{16}$ we proceed as follows: we take each element of the reduced set of $S_4^{15}$, extract its rectangular symmetry, and add one more full tile in all possible ways to the extracted tile combinations. Then we continue by filtering by symmetry and by the number of tiles in a quarter, and process the filtered combinations with the B&B algorithm, just like for $S_4^{18}$ and $S_4^{15}$. The tile combinations of $S_4^{17}$ are also processed in this incremental way, using the result combinations of $S_4^{16}$.

As a result of phase 1, we have all possible tile combinations of $S_4^k,\ k=15,16,17$,18 (apart from the filtered symmetry), that can be a subset of the tile combination of any optimal packing. Furthermore, the tile regions of each remaining combination are reduced so that they still certainly contain the subset of all optimal packing configurations. Note that it is not necessary to process the sets $S_4^k$ for $k<14$. The reason of this, on the one hand, is that such a tile combination cannot be a half of an optimal configuration. On the other hand, by decreasing *k*, the number of tile combinations to process will grow (until $k=12$), and at the same time we will be able to extract less and less information on the possible remaining regions.

#### Phase 2

In the second global phase the sets $S_8^{\ell ,r}$ are created and processed. We consider the cases $\ell \ge r$ only (note that this also filters out some symmetry). The processing is initiated by loading $S_4^k,\ k=15,\dots ,18$ into memory (also extracting the symmetric instances for all tile combinations), and sorting them for each *k* according to the bitset representation of their tile combination for fast searching. (Thus, in the sequel $S_4^k,\ k=15,\dots ,18$ will denote all remaining combinations *after extracting symmetry*.) The method given below is done first for $\ell =18,\ r=15$, then for $\ell =17,\ r=16$:

For each pairs of tile combinations $T_\ell \in S_4^\ell$ and $T_r \in S_4^r$ we create a tile combination $T \in S_8^{\ell ,r}$, after shifting $T_r$ to the right half square. *T* thus initially contains a tile pattern bitset and the corresponding remaining regions, both joined together from the respective data of $T_\ell$ and $T_r$. We process *T* with the following algorithm (observe that the algorithm starts with the cheaper filtering methods and continues with the more and more expensive ones):

1. If $T_\ell$ can be filtered out by *horizontal reflection*, then filter out *T*.
2. *Count the tiles of T in the horizontal half square sized regions* of the tiling (i.e., in the regions consisting of rows 1–4, 2–5, 3–6). If this count is greater than 18 in any such region, filter out *T* (by Theorem 7).
3. Next *count the tiles of T in the vertical half square sized regions* (i.e., the regions consisting of rows 2–5, 3–6, 4–7; note that the regions of columns 1–4 and 5–8 are the two input regions, so they do not have to be checked). If this count is greater than 18 in any such region, then filter out *T*.
4. Next *check the tile patterns* (the bitsets) in all the above *vertical half square sized regions*. If the tile count in any vertical half is between 15 and 18, then search for the corresponding tile pattern in $S_4^k,\ k=15,\dots ,18$. If the pattern is not found, filter out *T* by Corollary 1.
5. If *T* passed all tile count and tile pattern tests so far, then check the *remaining regions of the vertical half square sized regions*. If the tile count in any vertical half is between 15 and 18, then, by the previous step, we have the remaining tile regions of these halves stored in $S_4^k,\ k=15,\dots ,18$. In this case, by Theorem 5 we can update the respective tile regions of *T* by intersecting them with the remaining regions from $S_4^k$. (Of course, the latter regions are always shifted to the right column positions.) If during any intersection steps a tile region becomes empty, then filter out *T* by Corollary 1.
6. Finally, *process the remaining regions of T with the B&B algorithm* (using the same settings as in phase 1), to create the reduced $S_8^{\ell ,r}$.

The respective lines of Table 1 contains the computational details of phase 2. $|S|_{filt}$ again refers to the number of those combinations that survived the filtering process (steps 1 to 5 above), and were passed to the B&B search, and $|S|_{red}$ are the number of combinations remained after the B&B search. It is worth observing that both the filtering steps and the B&B algorithm contributed significantly to the reduction of the tile combinations. As a result of phase 2 we had 391 combinations left, which were merged into the initial $S_8^{33}$ for the next phase.

#### Phase 3

In the third, local refinement phase, we execute the B&B algorithm for all elements in $S_8^{33}$ one by one. There is no filtering in this phase, so here $|S|_{filt}$ just refers to the number of input tile combinations. The settings of the B&B algorithm are the following for this phase:

1. The maximum number of iterations is increased to 20 000, to let the algorithm run much longer than in the previous phases.
2. The method for selecting the subdivision direction is also changed: we choose that of the rectangle of the current box for subdivision that followed the index of the last split rectangle of this box (starting at index 1 for the first selection), and bisect it perpendicular to its larger side. This method is based on the observation that, due to the large number of iterations of the local refinement phase, some components may remain significantly larger than the others (e.g., those that are constrained by less neighboring points in the packing or those that contain a free point, see below). Hence selecting the component with the largest area can easily cause excessive (and unnecessary) subdivisions. Splitting with equal frequency in all components helps to reduce this effect.
3. The reduction rectangles of Algorithm 2 are computed by the method of Sect. 5.2 (the improved one with reduced amount of overestimation), designed for the refinement phase to reach higher precision. Since the number of remaining subproblems is already small at this point, the additional amount of extra processing time was not significant in overall.

The first step of the local refinement phase reduces the number of remaining tile combinations to four. To reach a precision of the enclosures that is close to double precision, two additional small tools are used:

First, since the algorithm runs long for the four remaining combinations, we need to do a simple clustering of the subboxes remaining in ${\mathcal {W}}$ and ${\mathcal {R}}$. This gathers the unnecessary split subboxes together, and also, it is done with the purpose of identifying nearby but separate optimal solutions (although in theory this is possible, for the present three problem instances we did not find such solutions).

The second tool we use at this point is to guess free points (that is, points in optimal configurations that can slightly move without affecting optimality) in the remaining regions. This tool was used, in a somewhat different form, also in [6]. In detail, we employ the following lemma:

##### Lemma 2

Let $I \subset \{1, \dots ,n\}$ be an index set, $({\varvec{x}},{\varvec{y}}) \subseteq [0,1]^{2n}$ be a box, $(x_i,y_i) \in ({\varvec{x}}_i,{\varvec{y}}_i)$ for all *I*, and let ${\overline{f}}$ be an upper bound of the global maximum of the point packing problem (with squared distances). Assume that

4 $$\begin{aligned} {\mathrm{inf}}((x_i-{\varvec{x}}_j)^2 + (y_i-{\varvec{y}}_j)^2) > {\overline{f}}&\quad \forall i \in I,\ \forall j \not \in I,\ \mathrm {and} \end{aligned}$$

5 $$\begin{aligned} (x_i-x_j)^2 + (y_i-y_j)^2 > {\overline{f}}&\quad \forall i, j \in I,\ i \ne j. \end{aligned}$$

If $\{(x^*_j,y^*_j) \in ({\varvec{x}}_j,{\varvec{y}}_j), j \not \in I\}$ are the components of any optimal point packing, then these components can be extended with $\{ (x_i,y_i),\ i \in I \}$ as free points, to get an optimal packing.

##### Proof

Consider an optimal packing $\{(x_i^*,y_i^*) \in ({\varvec{x}}_i,{\varvec{y}}_i),\ i=1,\dots ,n\}$ (with objective function value less than or equal to ${\overline{f}}$), and replace its *i*th components with $(x_i,y_i)$ for all $i \in I$. Then from (4) and (5) we have

$$\begin{aligned} (x_i-x^*_j)^2 + (y_i-y^*_j)^2> {\overline{f}}&\quad \forall i \in I, \forall j\ \not \in I,\ \mathrm {and} \\ (x_i-x_j)^2 + (y_i-y_j)^2 > {\overline{f}}&\quad \forall i, j \in I,\ i \ne j, \end{aligned}$$

which implies that $(x_i,y_i)$ can be slightly moved without affecting the objective function value, that is, optimality.$\square$

**Fig. 5:**
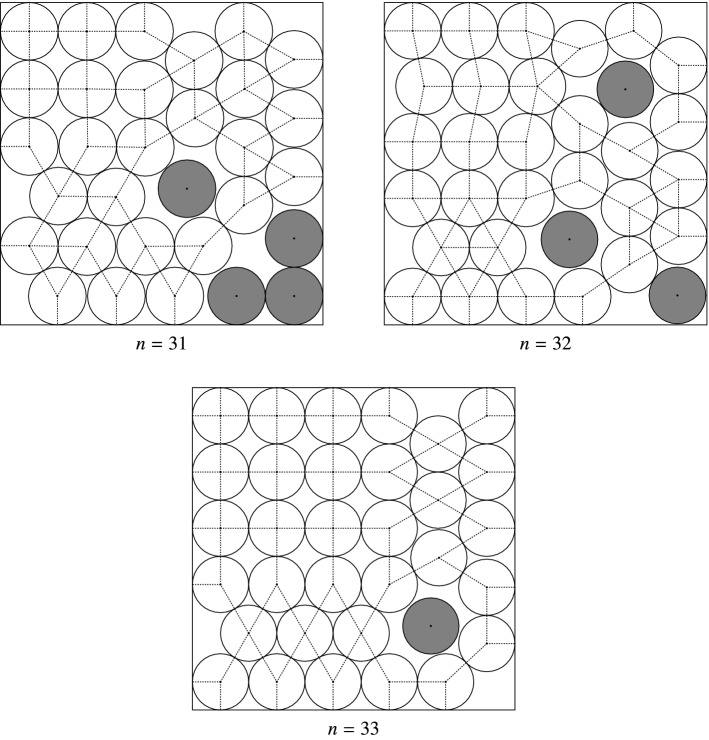
The optimal packings of $n=31,32,33$ circles

For $n=31,32,33$ we used the free points of the best known packings to create $\{ (x_i,y_i) \}$ (after applying symmetry transformations, when necessary), with 4, 3, and 1 free points, resp. (See also Fig. 5 in Sect. 7.5.) The interval-based verification of (4) and (5) was successful for all remaining $({\varvec{x}},{\varvec{y}})$ boxes at the end of the first step of phase 3 (with the exception of $n=31$, where one more refining step was needed to reduce the remain regions, so the free points have been detected after the second step of phase 3). The importance of the detection is that for the next local refinement step we can switch off bisecting the components in *I*, thus we can further accelerate the search and avoid unnecessary subdivisions.

For $n=33$, after clustering the results and identifying possible free points, in the second step of phase 3 we re-run the B&B search on the remaining 4 boxes (with the same settings) to increase the precision of the results. After analyzing the final four boxes, we recognized that the first and second one are from neighboring tile combinations. (Recall that every initial box passed to the B&B algorithm back in phase 1 was an individual tile combination.) The reason of this is that the possible locations of the (only) free point spread over two tile regions. The situation was the same for the third and fourth boxes. Furthermore, the first two and the last two boxes were proven to be symmetric to one of the diagonals of the square. (Note that since we filtered by the symmetry of the (rectangular) *tile regions*, it was not guaranteed that we filter out all symmetries in the square.) The optimal solution (taking the componentwise hull of the first two boxes) is given in Table 4, after transforming the results back to $[0,1]^2$. In the table underlines indicate the width of the enclosure intervals, that is, the precision of the result: if the width is $d \cdot 10^{-p}$ where $1 \le d < 10$, then the first *p* digits are underlined for both bounds. The table shows that it was possible to *provide very tight enclosures in all components* (except the free point) *with the precision of 13 to 15 digits*.

### Optimality proofs for $n=32,31$

The processes of finding all optimal packings of 32 and 31 points are very similar to the first instance. The only significant difference is the use of Theorem 6 in phase 1 (see also Remark 1): namely, when solving $n=32$, the initial sets of $S_4^k$ are the reduced ones computed for $n=33$ (if they are available). Similarly, when solving $n=31$, the input $S_4^k$ sets are the output sets $S_4^k$ computed for $n=32$ (if they are available). In both cases we can avoid a lot of repeated area reductions, though we estimate that this technique reduced the overall running time only by about 1 to 2%. The computational results of the two instances are shown in Tables 2 and 3.

After the end of phase 3, the number of result boxes was three for $n=32$, with symmetry properties similar to $n=33$: here the first two boxes come from neighboring tile combinations, due to a free point expanding in two neighboring tiles. The third box is diagonally symmetric to the first two, however, for this symmetric case the possible locations of the mentioned free point all fit into one tile. For $n=31$ we had only one box left. The enclosures of all global maximizers (apart from symmetry) are given in Tables 5 and 6. Both the computational complexity and the precision reached for the final results are similar to those obtained for $n=33$.

**Table 2:** Computational details of solving the packing problem of $n=32$ points

*S*	$|S|_{th}$ (approx.)	$|S|_{filt}$	$|S|_{red}$	CPUt (min.)
*Phase 1*				
$S_4^{18}$	$1.34\times 10^5$	5	1	0.0
$S_4^{14}$	$1.96\times 10^6$	457 340	292 900	380.8
$S_4^{15}$	$1.31\times 10^6$	153 593	113 833	165.3
$S_4^{16}$	$7.35\times 10^5$	37 825	22 391	36.7
$S_4^{17}$	$3.46\times 10^5$	2 935	1 177	2.2
*Phase 2*				
$S_8^{18,14}$	$2.64\times 10^{11}$	25 818	0	1.5
$S_8^{17,15}$	$4.53\times 10^{11}$	23 108 558	4 699	760.2
$S_8^{16,16}$	$5.41\times 10^{11}$	61 663 679	7 804	1 991.8
*Phase 3*				
$S_8^{32}$	$2.25\times 10^{12}$	12 503	3	295.9
$S_8^{32}$	$2.25\times 10^{12}$	3	3	10.4
$\sum$	–	85 462 259	442 811	3 644.8

**Table 3:** Computational details of solving the packing problem of $n=31$ points

*S*	$|S|_{th}$ (approx.)	$|S|_{filt}$	$|S|_{red}$	CPUt (min.)
*Phase 1*				
$S_4^{18}$	$1.34\times 10^5$	1	0	0.0
$S_4^{14}$	$1.96\times 10^6$	292 900	189 650	243.0
$S_4^{15}$	$1.31\times 10^6$	113 833	54 833	79.6
$S_4^{16}$	$7.35\times 10^5$	22 391	6 501	11.1
$S_4^{17}$	$3.46\times 10^5$	1 177	110	0.2
*Phase 2*				
$S_8^{17,14}$	$6.79\times 10^{11}$	1 024 058	0	36.2
$S_8^{16,15}$	$9.62\times 10^{11}$	36 228 141	1 984	1131.3
*Phase 3*				
$S_8^{31}$	$4.24\times 10^{12}$	1 984	1	40.0
$S_8^{31}$	$4.24\times 10^{12}$	1	2	8.6
$S_8^{31}$	$4.24\times 10^{12}$	2	1	4.3
$\sum$	–	37 684 488	253 082	1 554.3

**Table 4:** The enclosure of the global maximizers for packing 33 points

*i*	${\varvec{x}}_i$	${\varvec{y}}_i$
1	$[\underline{0.0000000000000}00,\underline{0.0000000000000}10]$,	$[\underline{0.00000000000000}0,\underline{0.00000000000000}6]$
2	$[\underline{0.105678610816}670,\underline{0.105678610816}785]$,	$[\underline{0.1830074237850}40,\underline{0.1830074237851}09]$
3	$[\underline{0.000000000000}000,\underline{0.000000000000}220]$,	$[\underline{0.366014847570}080,\underline{0.366014847570}212]$
4	$[\underline{0.00000000000000}0,\underline{0.00000000000000}5]$,	$[\underline{0.577343231713}343,\underline{0.577343231713}475]$
5	$[\underline{0.00000000000000}0,\underline{0.00000000000000}5]$,	$[\underline{0.788671615856}606,\underline{0.788671615856}738]$
6	$[\underline{0.00000000000000}0,\underline{0.00000000000000}5]$,	$[\underline{0.999999999999}869,\underline{1.000000000000}000]$
7	$[\underline{0.211357221633}341,\underline{0.211357221633}564]$,	$[\underline{0.0000000000000}00,\underline{0.0000000000000}66]$
8	$[\underline{0.2113572182268}59,\underline{0.2113572182268}69]$,	$[\underline{0.36601484953729}9,\underline{0.36601484953730}3]$
9	$[\underline{0.21132838414326}2,\underline{0.21132838414326}8]$,	$[\underline{0.57734323171347}1,\underline{0.57734323171347}5]$
10	$[\underline{0.21132838414326}2,\underline{0.21132838414326}9]$,	$[\underline{0.78867161585673}4,\underline{0.78867161585673}8]$
11	$[\underline{0.21132838414326}2,\underline{0.21132838414326}8]$,	$[\underline{0.99999999999999}7,\underline{1.00000000000000}0]$
12	$[\underline{0.317035829043}529,\underline{0.317035829043}645]$,	$[\underline{0.1830074257521}94,\underline{0.1830074257522}62]$
13	$[\underline{0.4227144364537}18,\underline{0.4227144364537}28]$,	$[\underline{0.00000000000000}0,\underline{0.00000000000000}3]$
14	$[\underline{0.422714429643}971,\underline{0.422714429644}197]$,	$[\underline{0.3660148554366}53,\underline{0.3660148554367}18]$
15	$[\underline{0.4226567682865}25,\underline{0.4226567682865}31]$,	$[\underline{0.5773432317134}09,\underline{0.5773432317134}75]$
16	$[\underline{0.4226567682865}25,\underline{0.4226567682865}32]$,	$[\underline{0.7886716158566}72,\underline{0.7886716158567}38]$
17	$[\underline{0.4226567682865}25,\underline{0.4226567682865}31]$,	$[\underline{0.9999999999999}35,\underline{1.0000000000000}00]$
18	$[\underline{0.528393037054}160,\underline{0.528393037054}275]$,	$[\underline{0.1830074296844}55,\underline{0.1830074296845}24]$
19	$[\underline{0.634071637654}601,\underline{0.634071637654}825]$,	$[\underline{0.000000000000}000,\underline{0.000000000000}129]$
20	$[\underline{0.6341713076398}21,\underline{0.6341713076398}36]$,	$[\underline{0.36595726821002}0,\underline{0.36595726821002}8]$
21	$[\underline{0.63398514456328}2,\underline{0.63398514456328}8]$,	$[\underline{0.57728557035602}0,\underline{0.57728557035602}9]$
22	$[\underline{0.63398515046269}7,\underline{0.63398515046270}3]$,	$[\underline{0.7886427817731}23,\underline{0.7886427817731}42]$
23	$[\underline{0.63398515242978}9,\underline{0.63398515242979}4]$,	$[\underline{0.99999999999999}2,\underline{1.00000000000000}0]$
24	$[\underline{0.845400021797}864,\underline{0.845400021798}088]$,	$[\underline{0.000000000000}000,\underline{0.000000000000}238]$
25	$[\underline{0.7}42408052211766,\underline{0.8}17880315336449]$,	$[\underline{0.1}84532761712745,\underline{0.2}56698005516485]$
26	$[\underline{0.820133056170}537,\underline{0.820133056170}612]$,	$[\underline{0.466346082467}272,\underline{0.466346082467}400]$
27	$[\underline{0.81699257424780}1,\underline{0.81699257424780}9]$,	$[\underline{0.6829641709564}60,\underline{0.6829641709564}71]$
28	$[\underline{0.81699257621488}9,\underline{0.81699257621489}7]$,	$[\underline{0.8943213891833}19,\underline{0.8943213891833}30]$
29	$[\underline{0.999999999999}778,\underline{1.000000000000}000]$,	$[\underline{0.144078217245}015,\underline{0.144078217245}254]$
30	$[\underline{0.999999999999}863,\underline{1.000000000000}000]$,	$[\underline{0.355406601388}278,\underline{0.355406601388}517]$
31	$[\underline{0.99999999999999}4,\underline{1.00000000000000}0]$,	$[\underline{0.5772855635462}64,\underline{0.5772855635462}83]$
32	$[\underline{0.99999999999999}7,\underline{1.00000000000000}0]$,	$[\underline{0.78864277836665}1,\underline{0.78864277836665}9]$
33	$[\underline{0.9999999999999}89,\underline{1.0000000000000}00]$,	$[\underline{0.9999999999999}82,\underline{1.0000000000000}00]$

Underlines indicate the width of the result intervals

**Table 5:** The enclosure of the global maximizers for packing 32 points

*i*	${\varvec{x}}_i$	${\varvec{y}}_i$
1	$[\underline{0.0000000000000}00,\underline{0.0000000000000}10]$,	$[\underline{0.0000000000000}00,\underline{0.0000000000000}10]$
2	$[\underline{0.1065872812949}34,\underline{0.1065872812949}48]$,	$[\underline{0.1846145866434}64,\underline{0.1846145866434}75]$
3	$[\underline{0.00000000000000}0,\underline{0.00000000000000}9]$,	$[\underline{0.3692291732869}29,\underline{0.3692291732869}42]$
4	$[\underline{0.0000000000000}00,\underline{0.0000000000000}21]$,	$[\underline{0.5824037358768}06,\underline{0.5824037358768}19]$
5	$[\underline{0.0429736452139}39,\underline{0.0429736452139}63]$,	$[\underline{0.7912018679384}00,\underline{0.7912018679384}10]$
6	$[\underline{0.0000000000000}00,\underline{0.0000000000000}18]$,	$[\underline{0.99999999999999}4,\underline{1.00000000000000}0]$
7	$[\underline{0.2131745625898}76,\underline{0.2131745625898}90]$,	$[\underline{0.0000000000000}00,\underline{0.0000000000000}14]$
8	$[\underline{0.2131745625898}76,\underline{0.2131745625898}90]$,	$[\underline{0.3692291732869}33,\underline{0.3692291732869}43]$
9	$[\underline{0.2131745625898}76,\underline{0.2131745625899}03]$,	$[\underline{0.5824037358768}09,\underline{0.5824037358768}19]$
10	$[\underline{0.2131745625898}76,\underline{0.2131745625899}01]$,	$[\underline{0.99999999999999}5,\underline{1.00000000000000}0]$
11	$[\underline{0.3197618438848}11,\underline{0.3197618438848}32]$,	$[\underline{0.1846145866434}65,\underline{0.1846145866434}80]$
12	$[\underline{0.2561482078038}18,\underline{0.2561482078038}39]$,	$[\underline{0.79120186793840}2,\underline{0.79120186793841}0]$
13	$[\underline{0.4263491251797}52,\underline{0.4263491251797}67]$,	$[\underline{0.00000000000000}0,\underline{0.00000000000000}8]$
14	$[\underline{0.4263491251797}52,\underline{0.4263491251797}87]$,	$[\underline{0.3692291732868}98,\underline{0.3692291732869}42]$
15	$[\underline{0.426349125179}752,\underline{0.426349125179}893]$,	$[\underline{0.582403735876}774,\underline{0.582403735876}818]$
16	$[\underline{0.4693227703936}95,\underline{0.4693227703937}16]$,	$[\underline{0.7912018679383}96,\underline{0.7912018679384}09]$
17	$[\underline{0.4263491251797}52,\underline{0.4263491251797}96]$,	$[\underline{0.9999999999999}90,\underline{1.0000000000000}00]$
18	$[\underline{0.6395236877696}29,\underline{0.6395236877696}44]$,	$[\underline{0.0000000000000}00,\underline{0.0000000000000}17]$
19	$[\underline{0.5}32936406474687,\underline{0.6}29399401634653]$,	$[\underline{0.1}84614586635930,\underline{0.2}28792625209860]$
20	$[\underline{0.6286416704729}41,\underline{0.6286416704729}80]$,	$[\underline{0.4364684494146}58,\underline{0.4364684494147}04]$
21	$[\underline{0.6287106033254}52,\underline{0.6287106033254}96]$,	$[\underline{0.6496430008593}54,\underline{0.6496430008593}99]$
22	$[\underline{0.628660434414}802,\underline{0.628660434415}328]$,	$[\underline{0.93281720241}7104,\underline{0.93281720241}8201]$
23	$[\underline{0.8153854133565}08,\underline{0.8153854133565}75]$,	$[\underline{0.1204825614309}68,\underline{0.1204825614310}63]$
24	$[\underline{0.8153854133564}87,\underline{0.8153854133565}37]$,	$[\underline{0.333657124020}851,\underline{0.333657124020}946]$
25	$[\underline{0.816410131219}076,\underline{0.816410131219}211]$,	$[\underline{0.548587167955}480,\underline{0.548587167955}710]$
26	$[\underline{0.7}88120834443415,\underline{0.8}15385413356685]$,	$[\underline{0.7}61667643841610,\underline{0.7}91176644707691]$
27	$[\underline{0.830971743649}852,\underline{0.830971743650}190]$,	$[\underline{0.999999999999}752,\underline{1.000000000000}000]$
28	$[\underline{0.99}1247138943389,\underline{1.00}0000000000000]$,	$[\underline{0.0}00000000000000,\underline{0.0}13895280136112]$
29	$[\underline{0.9999999999999}84,\underline{1.0000000000000}00]$,	$[\underline{0.2270698427259}62,\underline{0.2270698427259}88]$
30	$[\underline{0.9999999999999}88,\underline{1.0000000000000}00]$,	$[\underline{0.4402444053158}39,\underline{0.4402444053158}66]$
31	$[\underline{0.999999999999}790,\underline{1.000000000000}000]$,	$[\underline{0.656929930595}115,\underline{0.656929930595}554]$
32	$[\underline{0.999999999999}727,\underline{1.000000000000}000]$,	$[\underline{0.870104493184}992,\underline{0.870104493185}431]$

Underlines indicate the width of the result intervals

**Table 6:** The enclosure of the global maximizers for packing 31 points

*i*	${\varvec{x}}_i$	${\varvec{y}}_i$
1	$[\underline{0.1060708146559}16,\underline{0.1060708146560}01]$,	$[\underline{0.0000000000000}00,\underline{0.0000000000000}22]$
2	$[\underline{0.0000000000000}00,\underline{0.0000000000000}22]$,	$[\underline{0.1899363218792}92,\underline{0.1899363218793}25]$
3	$[\underline{0.1103465838237}57,\underline{0.1103465838238}38]$,	$[\underline{0.3774208693204}81,\underline{0.3774208693205}41]$
4	$[\underline{0.000000000000}000,\underline{0.000000000000}148]$,	$[\underline{0.5649054167616}64,\underline{0.5649054167617}52]$
5	$[\underline{0.0000000000000}00,\underline{0.0000000000000}36]$,	$[\underline{0.7824527083807}88,\underline{0.7824527083808}76]$
6	$[\underline{0.0000000000000}00,\underline{0.0000000000000}88]$,	$[\underline{0.9999999999999}13,\underline{1.0000000000000}00]$
7	$[\underline{0.217525087176}831,\underline{0.217525087177}171]$,	$[\underline{0.186828181031}627,\underline{0.186828181031}845]$
8	$[\underline{0.2206554324665}92,\underline{0.2206554324666}52]$,	$[\underline{0.5649276212040}19,\underline{0.5649276212040}45]$
9	$[\underline{0.2175472916191}24,\underline{0.2175472916191}60]$,	$[\underline{0.7824527083808}51,\underline{0.7824527083808}76]$
10	$[\underline{0.2175472916191}24,\underline{0.2175472916192}12]$,	$[\underline{0.9999999999999}75,\underline{1.0000000000000}00]$
11	$[\underline{0.328979359697}667,\underline{0.328979359698}338]$,	$[\underline{0.000000000000}000,\underline{0.000000000000}129]$
12	$[\underline{0.3278716710005}90,\underline{0.3278716710006}70]$,	$[\underline{0.3743127284729}90,\underline{0.3743127284730}58]$
13	$[\underline{0.439325943521}455,\underline{0.439325943521}771]$,	$[\underline{0.187484547441}183,\underline{0.187484547441}420]$
14	$[\underline{0.4381805196434}27,\underline{0.4381805196435}05]$,	$[\underline{0.5618194803564}98,\underline{0.5618194803565}73]$
15	$[\underline{0.4350723787959}55,\underline{0.4350723787960}05]$,	$[\underline{0.7793445675333}30,\underline{0.7793445675334}20]$
16	$[\underline{0.4350945832382}48,\underline{0.4350945832383}25]$,	$[\underline{0.999999999999}877,\underline{1.000000000000}000]$
17	$[\underline{0.549672527345}206,\underline{0.549672527345}397]$,	$[\underline{0.0000000000000}00,\underline{0.0000000000000}50]$
18	$[\underline{0.5}69997950979352,\underline{0.6}11243121634352]$,	$[\underline{0.3}88756547950396,\underline{0.4}30001290371418]$
19	$[\underline{0.6225791306794}61,\underline{0.6225791306795}21]$,	$[\underline{0.8896534161761}72,\underline{0.8896534161762}49]$
20	$[\underline{0.656865616334}467,\underline{0.656865616334}787]$,	$[\underline{0.189305218532}634,\underline{0.189305218532}913]$
21	$[\underline{0.6256872715269}44,\underline{0.6256872715270}02]$,	$[\underline{0.672128328999}314,\underline{0.672128328999}416]$
22	$[\underline{0.7}66888790693767,\underline{0.7}84360116798573]$,	$[\underline{0.0}00000000000000,\underline{0.0}28744823250302]$
23	$[\underline{0.810694781467}086,\underline{0.810694781467}316]$,	$[\underline{0.343134383665}284,\underline{0.343134383665}530]$
24	$[\underline{0.812515452558}638,\underline{0.812515452558}813]$,	$[\underline{0.560674056478}302,\underline{0.560674056478}535]$
25	$[\underline{0.813171818968}167,\underline{0.813171818968}404]$,	$[\underline{0.782474912822}919,\underline{0.782474912823}169]$
26	$[\underline{0.8100636781206}69,\underline{0.8100636781207}12]$,	$[\underline{0.9999999999999}85,\underline{1.0000000000000}00]$
27	$[\underline{0.9}76779422158677,\underline{1.0}00000000000000]$,	$[\underline{0.0}00000000000000,\underline{0.0}16475699215404]$
28	$[\underline{0.9}87877831772954,\underline{1.0}00000000000000]$,	$[\underline{0.2}16908432391140,\underline{0.2}32791951566360]$
29	$[\underline{0.9999999999999}58,\underline{1.0000000000000}00]$,	$[\underline{0.450327472654}608,\underline{0.450327472654}788]$
30	$[\underline{0.999999999999}868,\underline{1.000000000000}000]$,	$[\underline{0.671020640301}839,\underline{0.671020640302}304]$
31	$[\underline{0.9999999999999}78,\underline{1.0000000000000}00]$,	$[\underline{0.8939291853440}19,\underline{0.8939291853440}93]$

Underlines indicate the width of the result intervals

### Summary of results

The results of the previous two subsections are summarized as follows: *Let* $n \in \{31, 32, 33\}$. *Apart from the symmetries of the square, all globally optimal solutions of the problem of packing* *n* *points are located in the boxes given in* Tables 4, 5, and  6, *resp.* Except the components containing possibly free points and one component for $n=32$ with the precision of 12 digits, all enclosures are given to the precision of 13–15 digits.

Furthermore, from the result boxes we computed that *the enclosure of the global optimum of the problem of packing n points is*

$$\begin{aligned} \begin{array}{ll} f_{31}^*=[\underline{0.21754729161912}43,\underline{0.21754729161912}80], &{} \ w(f^*_{31}) \approx 4\cdot 10^{-15}, \\ f_{32}^*=[\underline{0.21317456258987}64,\underline{0.21317456258987}79], &{} \ w(f_{32}^*) \approx 1\cdot 10^{-15}, \\ f_{33}^*=[\underline{0.21132838414326}31,\underline{0.21132838414326}45], &{} \ w(f_{33}^*) \approx 1\cdot 10^{-15}, \end{array} \end{aligned}$$

resp., that is, the exact global optima differ from the currently best known function values by at most $w(f^*_n)$.

The total CPU time required for solving the three instances was approximately 26, 61, and 13 hours, resp., instead of the months of CPU time estimated by the earlier method [6]. Although it is very hard to compare the performance of the present and that of the former interval-based computer aided proofs (since different problems have been solved on different hardware-software architectures), below we give a rough estimate for the improvement: If we use the number of total tile combinations to represent the overall hardness of a problem instance, and take the time needed to solve $n=28,29,30$, we find that the new solution procedure tackled $n=31,32,33$ about 255, 93, and 165 times faster than it was predicted with the use of the former method. The speedup between the present hardware-software environment and that of in [6] (Intel P4 1400 MHz CPU and the Profil/BIAS interval library [17]) is about 2.5 for interval operations, the algorithms the most depending on (this number somewhat include the compiler improvements as well). Thus if we correct the above time ratios with this number, we obtain that *the performance of the new method was roughly 40 to 100 times better than that of the predecessor method*.

Figure 5 shows the optimal packings for $n=31,32,33$, transformed to the visually more attractive circle packing problem. (Due to the high precision enclosures the centers appears as points.) In the figure the shaded circles are the free ones. The center of two circles (or a center and a side of the square) is connected if their interval distance is so that they *may* touch each other in an optimal solution. On the other hand, if they are not connected, then they certainly do not touch each other in any optimal solution. The depicted packings are actually identical to those of the best found ones so far [13], so the displayed possible touchings are indeed touchings in those configurations.

## Conclusions and future work

In this work an interval based optimization method was presented for solving circle (point) packing problems. A similar former method was revisited and improved, and with the new algorithm the open problem instances $n=31,32,33$ have been solved to global optimality. High precision enclosures were given for all optimal solutions and the global optimum value in all three cases. The most important contributions that led to the success of the new method were the following:

- We introduced a new, mathematically rigorous representation of polygons, and presented a new area elimination method that resulted in a simpler implementation, easier proof of correctness, and faster source code than its predecessor.
- We implemented the key part of the area elimination method in two versions: a faster one with more overestimation, to be used in the initial phases of the proof, and a second one with reduced overestimation, to provide high precision final enclosures.
- For the global phase (devoted to reduce the number of tile combinations) we
  - Used advanced basic data structures (based on bitsets) for the fast manipulation and search of tile combinations;
  - Extended the previous tile reduction tools with two more, one for bounding the number of tiles in a subregion by an already known optimal packing of lower dimension, and one for utilizing the partial results of a higher problem instance when solving the previous one;
  - Employed more thorough symmetry filtering and tile pattern matching techniques than before;
  so that with these tools we were able to reduce the number of global phases from three to two, as compared to the former method.

It is strongly believed that with simple modifications of the current method, the instances $n=34,35$ can also be tackled with similar computing efforts. This would close the gap in the solved problem instances (up to now, the cases $2,\dots ,9, 14, 16, 25$, and 36 are solved by hand, and every other instance $n < 36$ except $n=34,35$ are solved on a computer), which would be a milestone in the half-century old history of solving these problems. Furthermore, the interval polygon structure is designed in such a way that it can be generalized for solving similar problems, for example point packing problems on the sphere.

## Data Availability

Data sharing not applicable to this article as no datasets were generated or analysed during the current study.

## References

[1] Szabó PG, Markót MC, Csendes T, Specht E, Casado LG, García I (2007). New Approaches to Circle Packing in a Square.

[2] de Groot, C., Monagan, M., Peikert, R. Würtz, D.: Packing circles in a square: review and new results. In P. Kall (ed.), System Modeling and Optimization (Proc. 15th IFIP Conf. Zürich, 1991), Lecture Notes in Control and Information Services 180, pp. 45–54 (1992)

[3] de Groot, C., Peikert, R., Würtz, D.: The optimal packing of ten equal circles in a square, IPS Research Report 90–12. ETH, Zürich (1990)

[4] Peikert R (1994). Dichteste Packungen von gleichen Kreisen in einem Quadrat. Elem. Math..

[5] Nurmela KJ, Östergård PRJ (1999). More optimal packings of equal circles in a square. Discret. Comput. Geom..

[6] Markót MC, Csendes T (2005). A new verified optimization technique for the “packing circles in a unit square" problems. SIAM J. Optim..

[7] Hansen E (1992). Global Optimization using Interval Analysis.

[8] Moore RE, Kearfott RB, Cloud MJ (2009). Introduction to Interval Analysis.

[9] Ratschek H, Rokne J (1988). New Computer Methods for Global Optimization.

[10] Markót MC (2000). An interval method to validate optimal solutions of the “packing circles in a unit square" problems. Central Eur. J. Oper. Res..

[11] Nurmela,K.J., Östergård, P.R.J. : Optimal packings of equal circles in a square. In Y. Alavi, D.R. Lick, and A. Schwenk (eds.): Combinatorics, Graph Theory, and Algorithms (Proc. 8th Quadrennial International Conference on Graph Theory, Combinatorics, Algorithms, and Applications), pp. 671–680 (1999)

[12] Markót MC, Csendes T (2006). A reliable area reduction technique for solving circle packing problems. Computing.

[13] http://www.packomania.com, maintained by Eckard Specht

[14] Hofschuster, W., Krämer, W.: C-XSC 2.0: A C++ library for extended scientific computing. In: Numerical Software with Result Verification, Lecture Notes in Computer Science 2991, pp. 15–35 (2004)

[15] Graham RL, Lubachevsky BD (1996). Repeated patterns of dense packings of equal disks in a square. Electron. J. Combin..

[16] Boll DW, Donovan J, Graham RL, Lubachevsky BD (2000). Improving dense packings of equal disks in a square. Electron. J. Comb..

[17] Knüppel, O.: PROFIL: Programmer’s runtime optimized fast interval library. Bericht 93.4., Technische Universität Hamburg-Harburg (1993)

